# Distinct Interactions of Arsenite and Cadmium with Metal Homeostasis and the Cellular Stress Response in Human Bronchial Epithelial Cells

**DOI:** 10.3390/ijms27177809

**Published:** 2026-08-31

**Authors:** Martin Link, Jana Kuhn, Marlene Parsdorfer, Andrea Hartwig

**Affiliations:** Department of Food Chemistry and Toxicology, Institute of Applied Biosciences (IAB), Karlsruhe Institute of Technology (KIT), 76131 Karlsruhe, Germany

**Keywords:** arsenite, cadmium, gene expression, protein expression, oxidative stress, metal homeostasis, shotgun proteomics

## Abstract

Although exposure to heavy metals such as cadmium and inorganic arsenic has declined in recent decades, they remain prevalent environmental contaminants in food, drinking water, and tobacco smoke. This study aimed to identify and compare molecular endpoints of arsenite and cadmium toxicity at the gene and protein expression levels, with a focus on metal homeostasis, the oxidative stress response and redox-regulated processes. Human bronchial epithelial cells (BEAS-2B) were used as an *in vitro* model. First, the appropriate exposure conditions were defined by assessing cytotoxicity and intracellular metal accumulation. The cells were then incubated with 1–10 µM NaAsO_2_ or 1–5 µM CdCl_2_ for 24 h, after which gene and protein expression were analyzed. When compared to arsenite, cadmium was a markedly stronger inducer of metallothionein (MT) expression, indicating distinct effects on metal homeostasis. Both metals induced a concentration-dependent upregulation of oxidative stress-related genes. Cadmium led to a stronger activation of NF-κB signaling, while arsenite decreased selenoprotein-associated gene expression and affected genes involved ferroptosis regulation. Differences in gene and protein expression patterns were also observed, suggesting that transcriptional responses do not necessarily translate into corresponding changes at the protein level after 24 h. These findings highlight shared and compound-specific mechanisms, as well as the importance of multi-level analyses.

## 1. Introduction

Environmental contaminants, such as heavy metals and metalloids, pose a potential risk to human health. Although daily exposure to heavy metals has decreased over the last few decades [1], occupational exposure, smoking, and environmental exposure are still considered toxicologically relevant [2]. Inorganic trivalent arsenic compounds and cadmium are especially critical (semi)metals in this context. The International Agency for Research on Cancer (IARC) has classified both as carcinogenic to humans, mainly based on lung cancer upon inhalative exposure in occupational settings [3]. Regarding environmental exposure towards cadmium, mostly by oral uptake, the most sensitive endpoint is nephrotoxicity, and a tolerable weekly intake (TWI) value of 2.5 µg/kg of body weight per week has been established [4]. In contrast, no safe intake level can be established for inorganic arsenic, which considerably elevates the risk of bladder, lung, and skin cancer following oral exposure in humans already at environmental exposure levels [5]. Since inorganic arsenic and cadmium both cause lung cancer, examining the toxicity of these metals in lung models is particularly relevant.

Divalent cadmium and trivalent arsenite ions are taken up by different mechanisms into human cells. Cadmium ions compete with other divalent metal ions at transport proteins such as the divalent metal transporter 1 (DMT1) [6], while arsenite is taken up via aquaglyceroporins (AQP), based on its structural similarity to glycerol [7]. Mechanistically, both metal compounds are known to induce oxidative stress, despite not being intrinsically redox-active. Therefore, indirect mechanisms that increase the level of reactive oxygen species are more relevant in both cases. Arsenite and cadmium both induce mitochondrial permeabilization, inhibit antioxidant enzymes, and release redox-active metal ions from proteins, thereby promoting oxidative stress. One particularly relevant proposed mechanism for arsenite involves the stimulation of nicotinamide adenine dinucleotide phosphate oxidase (NADPH oxidase, NOX), which leads to the generation of superoxide anions and peroxynitrite [8,9]. Due to their strong affinity for thiol groups, cysteine-containing proteins are specific targets of both arsenite and cadmium. One mechanism of toxicity involves displacing essential trace elements, such as zinc, from zinc-binding domains of respective proteins [10,11]. This can result in changes to their DNA-binding capacity, resulting for example in inhibition of poly(ADP-ribose) polymerase 1 (PARP1) activity at particularly low concentrations [12].

Both the binding to and the oxidation of cysteine residues in proteins can affect redox-sensitive signaling pathways and transcription factors. For example, the oxidation of the redox-sensitive cysteine residues of kelch-like ECH-associated protein 1 (KEAP1) releases nuclear factor erythroid 2-related factor 2 (NRF2). NRF2 then translocates into the nucleus and initiates the expression of genes related to the oxidative stress response and the antioxidant defense system [13]. Other transcription factors and signaling pathways have also been described as being redox-regulated, including nuclear factor ‘kappa-light-chain-enhancer’ of activated B-cells (NF-κB), hypoxia-inducible factor 1α (HIF-1α), and forkhead box O (FOXO) family proteins [13,14].

The mechanisms underlying the toxicity of both metal compounds have been studied for many years, but they are still not fully understood. Different studies have examined changes in gene and protein expression after treating BEAS-2B cells with arsenite or cadmium. Regarding the impact of cadmium on gene expression, mostly higher concentrations of 5–10 µM [15] or 20–60 µM [16] were applied. Another study compared global gene expression changes after a four hour incubation period with cadmium and arsenite, and demonstrated that there were only a few expression changes among the 1200 genes examined [17]. For arsenite, Chilakapati et al. conducted a global gene expression analysis within the µM concentration range. They identified hundreds of altered gene expressions after a 24 h incubation period with arsenite. While this study provides a detailed description of many pathways affected at two arsenite concentrations, no follow-up study on the protein expression level was performed [18].

Regarding proteomics, methods have been optimized for many years to enable increasingly sensitive measurements. Using stable isotope labeling by amino acids in cell culture (SILAC), arsenite treatment studies have been conducted in various cell models [19]. In BEAS-2B cells specifically, protein expression following 24 h of arsenite treatment was investigated using isobaric tags for relative and absolute quantification (iTRAQ) [20].

In the present study, we analyzed and compared the cellular uptake and expression of specific genes and proteins after incubating BEAS-2B cells for 24 h with sub- and low cytotoxic concentrations of arsenite and cadmium, starting at 1 µM for both compounds. Specifically, we focused on genes and proteins associated with metal homeostasis, oxidative stress response, and cellular redox homeostasis in BEAS-2B cells, paying close attention to redox-sensitive transcription factors. We identified common and distinct interactions with specific molecular pathways, providing new insight into cellular reactions in the low concentration ranges of both (semi)metals.

## 2. Results and Discussion

### 2.1. Cytotoxicity

**Figure 1:**
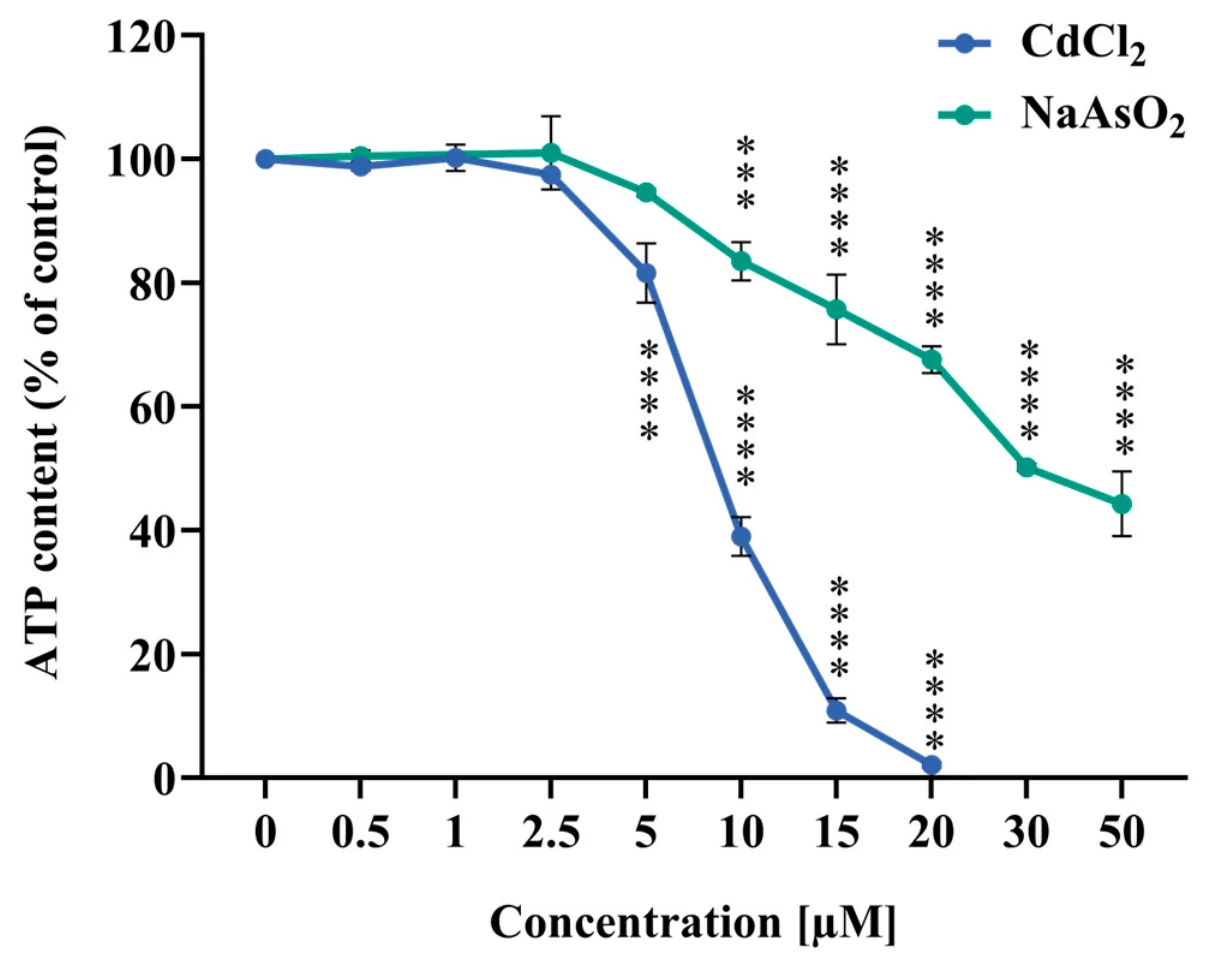
Intracellular ATP content of BEAS-2B cells as an indicator of cell viability after treatment with NaAsO_2_ (green) or CdCl_2_ (blue) for 24 h. Depicted are mean values ± standard deviation (SD) from three independent experiments, each comprising triplicate parallel cell cultures. A one-way ANOVA and Dunnett’s post hoc test were performed for statistical analysis, comparing the results to the untreated controls (*** *p* ≤ 0.001, **** *p* ≤ 0.0001).

The cytotoxicity of sodium arsenite (NaAsO_2_) and cadmium chloride (CdCl_2_) was evaluated to determine suitable incubation concentrations for subsequent experiments (Figure 1). BEAS-2B cells were treated with both metal compounds for 24 h. Then, the ATP content was measured using the CellTiterGlo^®^ luminescent cell viability assay (Promega). The ATP content was compared to that of untreated samples to serve as a measure of cell viability.

The results showed no evidence of cytotoxicity from either metal compound at concentrations up to 2.5 µM after 24 h of incubation. A small but significant decrease in ATP content was observed at 5 µM of CdCl_2_ (82%) and at 10 µM of NaAsO_2_ (84%). At 20 µM CdCl_2_, ATP levels decreased to 2%, whereas the same incubation concentration of NaAsO_2_ led to a decrease in ATP content to 64% compared to untreated controls. In general, CdCl_2_ exhibited higher cytotoxicity in BEAS-2B cells. The cytotoxicity of arsenite and cadmium has been studied previously using various test systems, and the results presented here are largely consistent with those reported in the literature [15,21]. The higher cytotoxicity of cadmium can be attributed to several factors, including increased induction of apoptosis and mitochondrial damage [16]. To obtain more mechanistic information, metal concentrations in the non- and low-cytotoxic range, up to 10 µM for NaAsO_2_ and 5 µM for CdCl_2_, were used in subsequent experiments.

### 2.2. Time-Dependent Cellular Uptake

**Figure 2:**
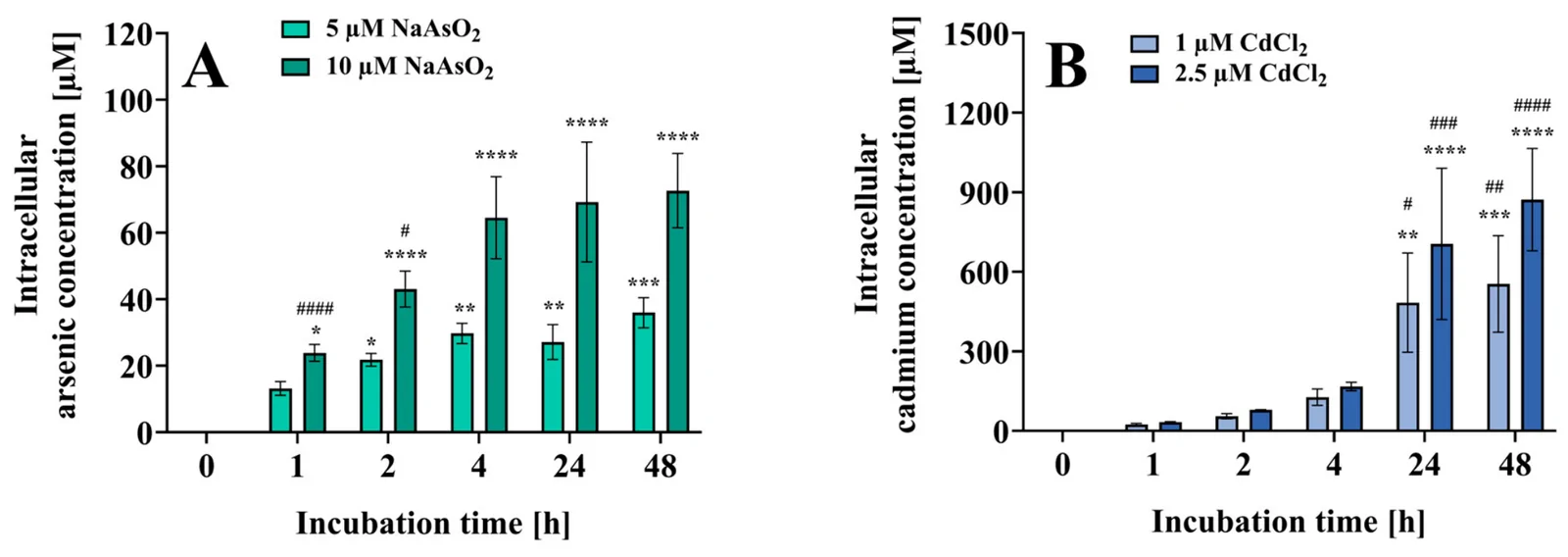
Time-dependent cellular uptake of arsenic (**A**) and cadmium (**B**) by BEAS-2B cells. Metal concentrations were analyzed using GF-AAS. The results are shown as the mean ± SD of three independent experiments, each comprising duplicate parallel cell cultures and three AAS measurements per sample. A two-way ANOVA with a Tukey’s post hoc test was performed for statistical analysis comparing the results to both untreated controls (* *p* ≤ 0.05, ** *p* ≤ 0.01, *** *p* ≤ 0.001, **** *p* ≤ 0.0001) and the 4 h treatment (^#^ *p* ≤ 0.05, ^##^ *p* ≤ 0.01, ^###^ *p* ≤ 0.001, ^####^ *p* ≤ 0.0001).

To select an appropriate incubation time for subsequent experiments, the time-dependent cellular uptake of arsenite and cadmium was assessed. Graphite furnace atomic absorption spectroscopy (GF-AAS) was used to determine the intracellular concentrations of arsenic and cadmium based on cell counts and average cell volumes (Figure 2A,B).

The basal cellular arsenic concentration was found to be 0.07 µM. Following arsenite treatment, time-dependent uptake was observed for up to 4 h, as demonstrated by statistical analyses comparing the 24 and 48 h exposures to the 4 h exposure. After 4 h, exposure to 5 µM and 10 µM arsenite resulted in intracellular arsenic concentrations of 30 µM and 65 µM, respectively. These results demonstrate that cells clearly accumulate arsenite in a time- and dose-dependent manner, reaching a plateau after approximately 4 h. Similar results have been reported for primary rat astrocytes. The authors observed concentration-dependent arsenic accumulation, reaching also a plateau after 4 h of arsenite treatment. Similar to our results, an approximately fivefold increase in intracellular arsenic concentration was reached [22]. This finding could be explained by an equilibrium between arsenite uptake and the excretion of arsenite, glutathione (GSH) adducts, or metabolites, primarily via multidrug resistance-associated proteins or bidirectional AQPs [7]. However, the metabolism of arsenite to trivalent or pentavalent monomethylarsonic acid (MMA) or dimethylarsinic acid (DMA) is less pronounced in bronchial epithelial cells than in hepatocytes [23], suggesting that this mechanism may not have contributed significantly to the results of this study.

The basal cellular cadmium concentration was 0.3 µM, which is about four times higher than the basal arsenic concentration. Cadmium uptake in BEAS-2B cells after CdCl_2_ treatment was dose- and time-dependent. Even at lower exposure concentrations, intracellular cadmium accumulation was far higher than arsenite accumulation, showing a considerable increase also between 4 h and longer incubation times. For instance, the cadmium concentration increased markedly from 167 µM to 705 µM between 4 and 24 h upon exposure to 2.5 µM CdCl_2_, reaching 900 µM after 48 h, which is equivalent to 3000-fold accumulation. A similar effect was also observed at the lower incubation concentration of 1 µM. At concentrations with comparable cytotoxicity, 5 µM NaAsO_2_ and 2.5 µM CdCl_2_, the cellular cadmium accumulation exceeded that of arsenite to a great extent, especially after longer incubation times.

Cadmium ions can enter cells through various divalent ion channels, including the DMT1 and zinc- or calcium-transport proteins [6]. Furthermore, cadmium strongly induces the expression of various metallothioneins (MTs) and exhibits a high binding affinity for these proteins [24]. One study on rats showed that after 8 h, most of the cellular cadmium was bound to MTs [25]. The substantial increase in intracellular cadmium levels between 4 and 24 h could therefore be explained by MT induction during this period. This was investigated in more detail in subsequent experiments. After 24 h, both metal compounds had been adequately taken up by the cells. Since protein expression is a downstream process of gene expression, a 24 h incubation period was selected to compare the two endpoints for both arsenite and cadmium.

### 2.3. Gene Expression Analysis

**Figure 3:**
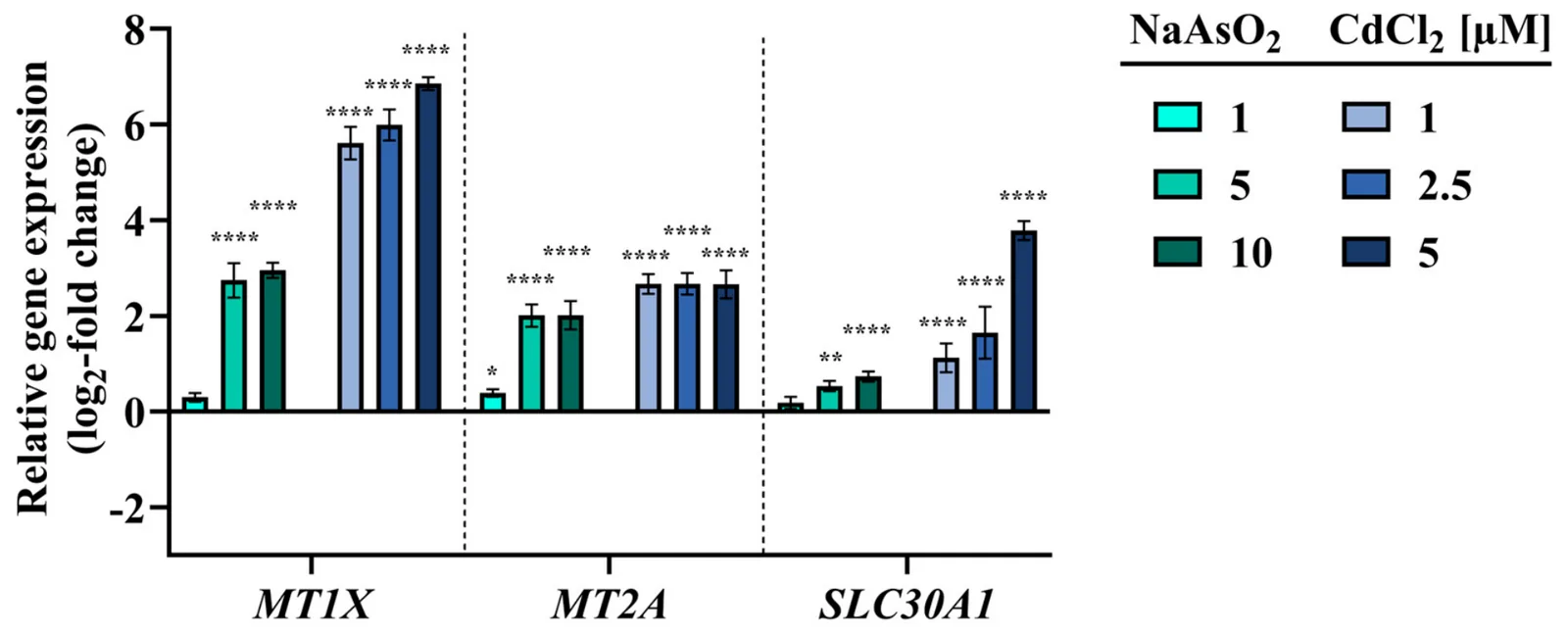
Effects of NaAsO_2_ and CdCl_2_ on the mRNA expression of genes related to metal homeostasis. BEAS-2B cells were incubated with arsenite or cadmium for 24 h. Relative gene expression (log_2_-fold change) is shown as the mean ± SD of three independent experiments. each comprising duplicate parallel cell cultures and two PCR measurements per sample. Statistical analysis was performed using a two-way ANOVA with a Dunnett’s post hoc test compared to the untreated control group (* *p* ≤ 0.05, ** *p* ≤ 0.01, **** *p* ≤ 0.0001).

**Figure 4:**
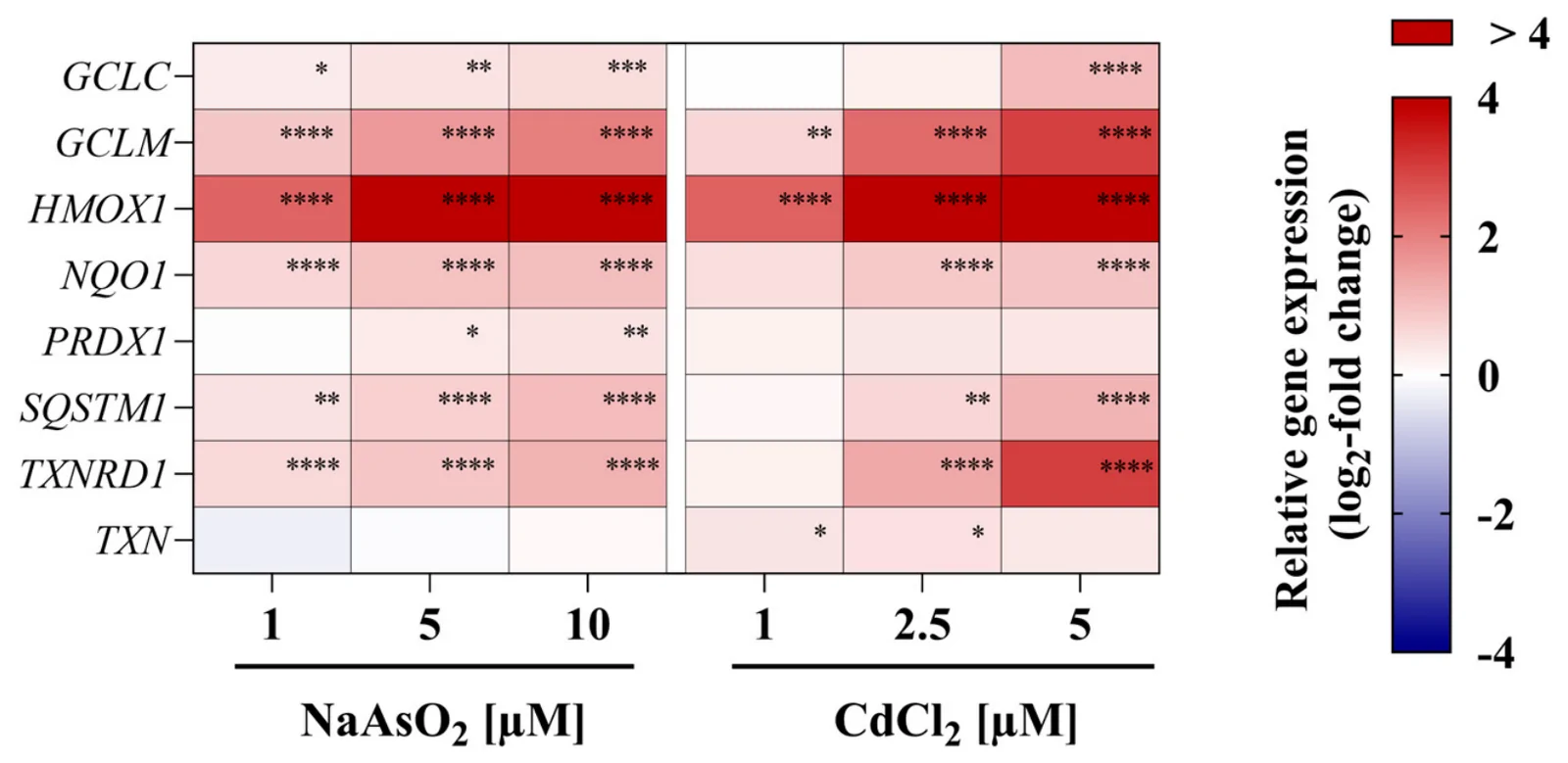
Effects of NaAsO_2_ and CdCl_2_ on NRF2-induced gene expression. BEAS-2B cells were incubated with arsenite or cadmium for 24 h. Relative gene expression (log_2_-fold change) is shown as the mean ± SD of three independent experiments. each comprising duplicate parallel cell cultures and two PCR measurements per sample. A two-way ANOVA with a Dunnett’s post hoc test was performed for statistical analysis compared to the untreated controls (* *p* ≤ 0.05, ** *p* ≤ 0.01, *** *p* ≤ 0.001, **** *p* ≤ 0.0001).

**Figure 5:**
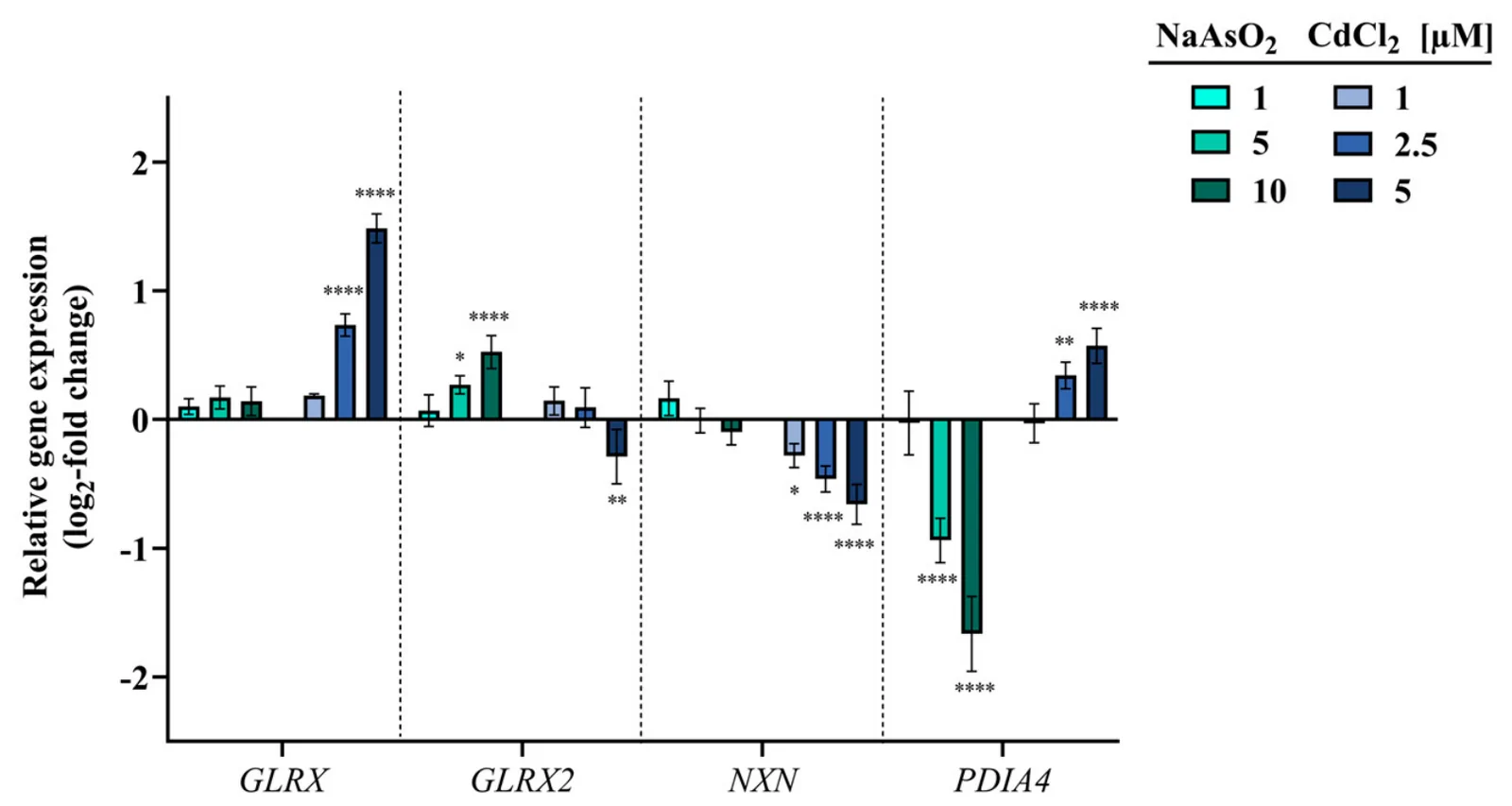
Effects of NaAsO_2_ and CdCl_2_ on the mRNA expression of genes related to redox regulation. BEAS-2B cells were incubated with arsenite or cadmium for 24 h. Relative gene expression (log_2_-fold change) is shown as the mean ± SD of three independent experiments, each comprising duplicate parallel cell cultures and two PCR measurements per sample. A two-way ANOVA with a Dunnett’s post hoc test was performed for statistical analysis compared to the untreated controls (* *p* ≤ 0.05, ** *p* ≤ 0.01, **** *p* ≤ 0.0001).

**Figure 6:**
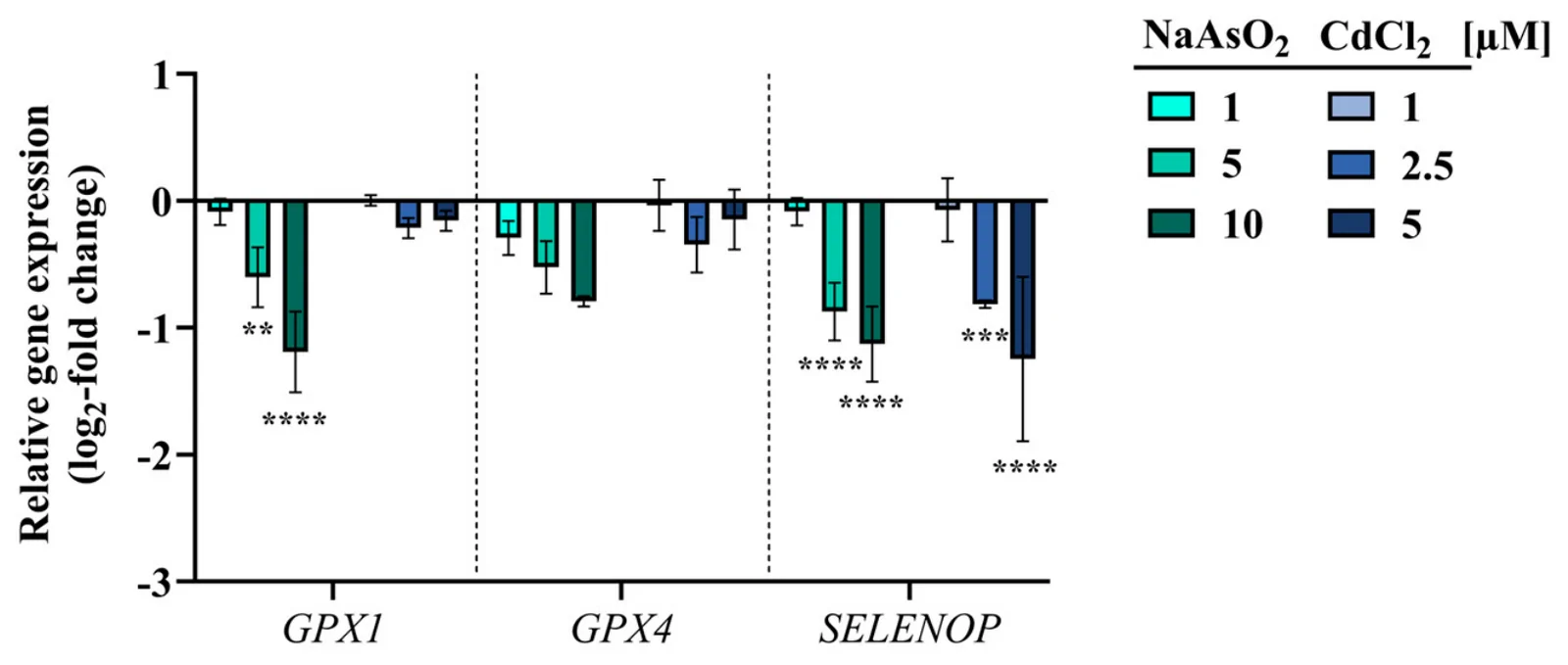
Effects of NaAsO_2_ and CdCl_2_ on the mRNA expression of selenium protein-related genes. BEAS-2B cells were incubated with arsenite or cadmium for 24 h. Relative gene expression (log_2_-fold change) is shown as the mean ± SD derived from three independent experiments, each comprising duplicate parallel cell cultures and two PCR measurements per sample. *GPX4* was analyzed in two independent experiments, and deviations are shown as Range/2. A two-way ANOVA with a Dunnett’s post hoc test was performed for statistical analysis compared to untreated controls (** *p* ≤ 0.01, *** *p* ≤ 0.001, **** *p* ≤ 0.0001).

**Figure 7:**
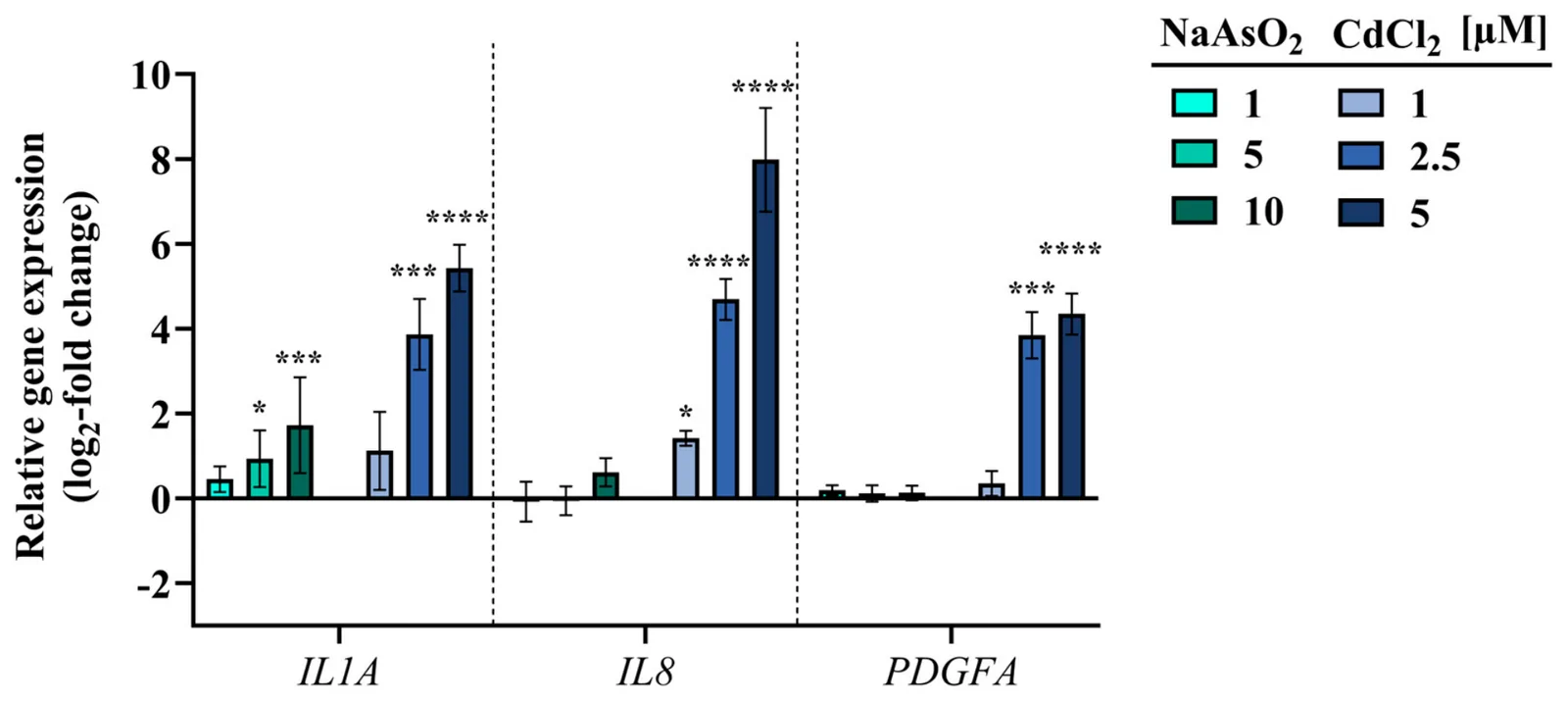
Effects of NaAsO_2_ and CdCl_2_ on the expression of genes encoding inflammation and fibrosis markers. BEAS-2B cells were incubated with arsenite or cadmium for 24 h. Relative gene expression (log_2_-fold change) is shown as the mean ± SD derived from three independent experiments, each comprising duplicate parallel cell cultures and two PCR measurements per sample. A two-way ANOVA with a Dunnett’s post hoc test was performed for statistical analysis compared to the untreated controls (* *p* ≤ 0.05, *** *p* ≤ 0.001, **** *p* ≤ 0.0001).

A high-throughput RT-qPCR approach was used to analyze the impact on the gene expression profiles of BEAS-2B cells after 24 h of arsenite or cadmium treatment. The expression of 95 genes, primarily associated with metal homeostasis, the oxidative stress response, redox regulation, and redox signaling, was analyzed. This method has been previously established and described by our group [15] and was applied in this study with modifications in the selected gene set (see Supplementary Table S1). Relative gene expression was analyzed as a log_2_-fold change by comparing treated samples to untreated controls. A relevant change in transcript levels of the respective genes was considered to be a reduction of at least 50% (log_2_-fold change ≤ −1) or a 2-fold increase (log_2_-fold change ≥ 1). Additionally, concentration-dependent trends were also taken into account. Some of the most pronounced effects are shown in Figure 3, Figure 4, Figure 5, Figure 6 and Figure 7. A summary of all analyzed gene expression data is presented in the Supplementary Table S2.

Regarding the expression of genes related to metal homeostasis, arsenite and cadmium exerted different pronounced effects, as shown in Figure 3.

After 24 h of incubation, even the lowest exposure concentration of 1 µM CdCl_2_ induced substantial increases in the expression of *MT1X* and *MT2A*, which encode two MT isoforms. *MT1X* mRNA levels increased about 49-fold, and those of *MT2A* about 6.3-fold. The expression of these genes did not (*MT2A*) or only slightly (*MT1X*) increase with higher incubation concentrations, suggesting saturation of MT expression. Gene expression of *SLC30A1*, which encodes the zinc transporter 1 (ZNT1), increased significantly in response to cadmium in a concentration-dependent manner, even at the lowest applied concentration. In contrast, exposure to 1 µM NaAsO_2_ had only minor effects on either MT-coding gene. At higher arsenite concentrations, *MT1X* expression reached a plateau at an approximately 3-fold increase, while *MT2A* expression increased by about 2-fold. Also, only a slight induction in *SCL30A1* expression was observed at higher arsenite concentrations. Therefore, cadmium exposure resulted in a stronger induction of genes related to metal homeostasis compared with arsenite at the concentrations investigated. This difference may be attributed, at least in part, to the substantially higher intracellular accumulation of cadmium after 24 h, as shown in Figure 2. Previous studies have shown that both arsenite and cadmium can bind to zinc-binding structures, resulting in the release of zinc ions, which in turn activate the metal regulatory transcription factor 1 (MTF-1) [26,27]. Due to its high affinity for MTs, cadmium has been reported to stimulate MT expression approximately eight times more potently than zinc does [28]. Furthermore, in astrocytes, cadmium has been shown to activate MTF-1-induced gene expression, including MT encoding genes and *SLC30A1*, eight times more effectively when compared to equitoxic concentrations of arsenite [29]. These data are consistent with those presented in the present study and correlate with the time-dependent intracellular uptake data, indicating a higher cadmium accumulation through MT induction compared to arsenite treatment.

NRF2 is a redox-sensitive transcription factor involved in the regulation of oxidative stress related genes [13]. Comparing the impact on genes regulated by NRF2, both metal exposures led to similar expression patterns, as shown in Figure 4.

Interestingly, in case of arsenite, almost all genes were already significantly up-regulated at the lowest exposure concentration, whereas cadmium exposure primarily induced up-regulation starting at the medium exposure level. Nevertheless, in both cases, starting at 1 µM, the most pronounced effect was observed for *HMOX1*, which encodes heme oxygenase 1, with a 5-fold increase at the lowest arsenite concentration and a 59-fold increase at the highest concentration analyzed. A 145-fold increase in *HMOX1* expression was detected at 5 µM CdCl_2_. Previous studies have already described *HMOX1* as a sensitive marker of the oxidative stress response to arsenite or cadmium exposure [15,18]. Notably, genes encoding the catalytic and modified subunits of glutamate-cysteine ligase (*GCLC* and *GCLM*) exhibited distinct expression patterns in response to metal exposure. At the medium dose of arsenite, *GCLC* transcript levels increased by a factor of 1.3, while *GCLM* levels increased by a factor of about 3. Cadmium incubation produced comparable but more pronounced effects. Thompson et al. observed that both genes were induced in mouse fibroblasts after four hours of treatment with 10 µM arsenite. However, both mRNA levels decreased thereafter, while *GCLM* mRNA appeared more stable [30]. Consistent with this finding, changes were still detectable in our study after 24 h. These data suggest that arsenite and cadmium may activate NRF2-dependent signaling, as indicated by the altered expression of NRF2 target genes. In this process, cysteine residues of the NRF2 inhibitor KEAP1 are oxidized, leading to conformational changes and thereby activating the transcription factor NRF2 [31]. Although trivalent arsenite and divalent cadmium ions are not considered directly redox-active, oxidative stress is promoted indirectly by inhibiting the antioxidant defense or by leading to mitochondrial dysfunction [8,9].

Figure 5 shows a more detailed examination of additional genes involved in cellular redox regulation whose transcriptional regulation is not fully understood.

In general, the effects were less pronounced than those of the gene expression shown in Figure 4. Notably, CdCl_2_ induced *GLRX* expression (which encodes glutaredoxin-1) in a concentration-dependent manner, increasing expression by up to 2.8-fold compared to untreated samples. These results may be due to the activation of NF-κB signaling, as has been suggested for *GLRX* expression in murine lung epithelial cells [32].

Interestingly, 24 h cadmium exposure did not affect the mRNA level of *GLRX2*, a mitochondrial glutaredoxin family member. The expression of *NXN*, which encodes the redox mediator nucleoredoxin, was slightly repressed by cadmium in a concentration-dependent manner. Arsenite, however, did not affect *GLRX* or *NXN* expression, and only a slight induction of *GLRX2* was observed, indicating differences in NF-κB activation.

A more pronounced effect was observed in the case of *PDIA4*, which encodes protein disulfide isomerase family A member 4. Contrary effects were observed for cadmium and arsenite, however. In general, protein disulfide isomerases (PDIs) are primarily expressed in the endoplasmic reticulum (ER) and are involved in forming disulfide bonds in newly synthesized proteins after translation [33]. Furthermore, *PDIA4* is induced by ER stress. As a structural component involved in redox-regulation, PDIA4 contains three redox-active thioredoxin domains [33]. The moderate induction of *PDIA4* gene expression observed in the present study after cadmium exposure could be an adaptation to ER stress. This finding is consistent with previous studies, demonstrating the induction of ER stress-associated genes. However, no data on *PDIA4* expression were obtained [34]. Additionally, it has been reported that cadmium promotes ER stress and activates the unfolded protein response (UPR) by binding to thiol groups and indirectly inducing oxidative stress, which leads to protein misfolding. Furthermore, cadmium significantly disrupts calcium homeostasis, especially in the ER. In this case, the UPR usually has a stronger pro-apoptotic effect [35]. While the results obtained for cadmium exposure therefore fit into the general picture of ER stress induction, the results obtained for arsenite exposure are not as easily explained. Arsenite treatment resulted in a dose-dependent reduction in *PDIA4* expression down to 30% compared to the control. This seems to contradict the activation of the URP response by arsenite, even though this has been reported in the literature [36]. Nevertheless, PDIA4 plays a role not only in the ER stress response but also in the regulation of ferroptosis. Thus, Kang et al. observed the downregulation of PDIA4 protein expression following salinomycin exposure in renal carcinoma cells, resulting in increased sensitivity to ferroptosis [37]. In support of this potential interpretation of our results, previous studies have described arsenite-induced ferroptosis in murine lung epithelial cells [38]. Although cadmium has also been reported to induce ferroptosis, particularly at higher concentrations [39], our data did not support this observation and provide new insights into the regulation and sensitivity of *PDIA4* gene expression upon metal exposure.

Decreased expression of genes encoding specific selenoproteins, including glutathione peroxidases 1 and 4 (*GPX1* and *GPX4*) as well as selenoprotein P (*SELENOP*) was also observed after arsenite treatment. Respective gene expression data are shown in Figure 6.

The selenoprotein GPX4 is an antioxidant that plays an important role in inhibiting ferroptosis. Our data demonstrate a concentration-dependent decrease in *GPX4* following arsenite treatment, which is consistent with previous reports on 10 µM arsenic(III)oxide treatment in human neuroblastoma cells [40]. Taken together with the down-regulation of *PDIA4* expression described above, these observations provide further support for a potential involvement of ferroptosis in arsenite-induced cellular responses. Arsenite also decreased *GPX1* mRNA levels by 56% at 10 µM, confirming previous results obtained in human liver cells [41]. One possible explanation is the formation of [(GS)_2_AsSe]^−^ complexes, as suggested in [42]. However, cadmium did not affect the expression of the *GPX1* and *GPX4* genes, even though the formation of cadmium-selenium complexes has been described as well [42]. Therefore, our findings suggest that arsenite may impact intracellular selenium homeostasis more strongly than cadmium. Within the selenoprotein hierarchy, *GPX1* gene expression is highly sensitive to selenium deficiency, resulting in decreased mRNA levels in different cell lines [43]. Interestingly, the concentration-dependent decrease in *SELENOP* expression was comparable between the two metals. Similar reductions in *SELENOP* mRNA and SELENOP protein levels were observed in HepG2 cells after pharmacological activation of NRF2 signaling [44]. Consequently, the induction of NRF2 by arsenite and cadmium (as shown in Figure 4) could lead to reduced *SELENOP* transcription.

Furthermore, the impact of both metal compounds on inflammatory gene expression is shown in Figure 7.

After 24 h of treatment with 2.5 or 5 µM CdCl_2_, we observed clear increases in *IL1A*, *IL8*, and *PDGFA* expression. In contrast, arsenite did not affect *IL8* or *PDGFA* expression, and its effect on *IL1A* induction was less pronounced when compared to cadmium treatment.

The expression of inflammatory genes is primarily regulated by the redox-sensitive NF-κB signaling pathway [13]. Thus, cadmium appears to strongly influence NF-κB-associated signaling. This is also indicated by the altered expression of NF-κB target genes such as *GLRX* (Figure 5). Studies have shown that even low concentrations of cadmium can activate this pathway [45]. The opposite may be true for arsenite. Arsenite binds to Cys^179^ in the α and β subunits of the IκB kinase (IKK) complex, thereby inhibiting its kinase activity. As a consequence, phosphorylation and subsequent degradation of IκB are prevented, which inhibits NF-κB activation [46]. To the best of our knowledge, this is the first study to demonstrate the induction of *PDGFA* growth factor gene expression following cadmium treatment in BEAS-2B cells. In human breast cancer cells, mRNA levels increased after three hours but returned to baseline levels after 24 h [47]. The mechanisms are not yet fully understood, but *PDGFA* is thought to be regulated by NRF2, which recruits specificity protein 1 (SP1) to the promoter region [48]. As shown in Figure 4, the effects of cadmium were consistent with NRF2-associated signaling activation. Notably, however, arsenite did not induce *PDGFA* expression despite pronounced changes in the expression of other NRF2 target genes.

### 2.4. Global Protein Expression Analysis—Shotgun Proteomics with Data-Dependent Acquisition

**Figure 8:**
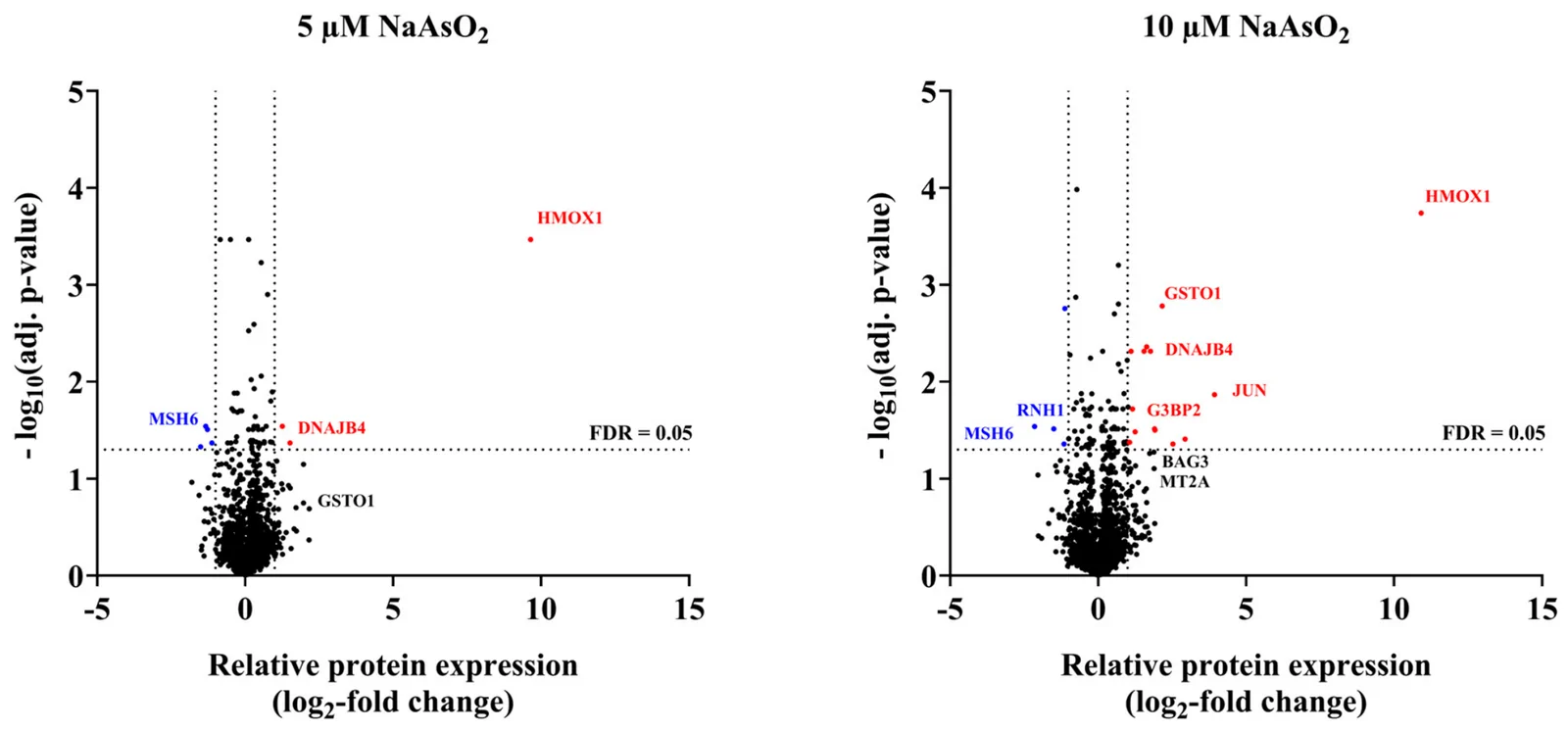
Effects of NaAsO_2_ on protein expression. BEAS-2B cells were incubated with 5 µM and 10 µM arsenite for 24 h. The relative protein expression is shown as the mean of three independent experiments, each comprising triplicate parallel cell cultures and one LC-MS injection per sample. Statistical significance compared to untreated controls was assessed by a two-sided Student’s *t*-test with Benjamini–Hochberg correction for multiple testing (false discovery rate (FDR) ≤ 0.05). Proteins showing significant and relevant changes in abundance are highlighted in blue when reduced by at least 50% and in red when increased at least two-fold relative to the control. Black dots indicate no significant and/or relevant changes in protein expression.

Next, we examined the effects of arsenite and cadmium on the expression of cellular proteins in BEAS-2B cells, applying a shotgun proteomics approach with data-dependent acquisition and label-free quantification. The results are presented as volcano plots in Figure 8 for the medium and highest concentrations. The volcano plot for the 1 µM metal incubation is shown in Figure S1 in the Supplementary Materials. In this chapter, only a selection of the most abundant and significantly changed proteins is discussed. To ensure clarity and consistency, proteins are referred to by their corresponding gene names throughout the following chapter. Full protein names and UniProt identifiers are provided in Supplementary Table S3.

**Figure 9:**
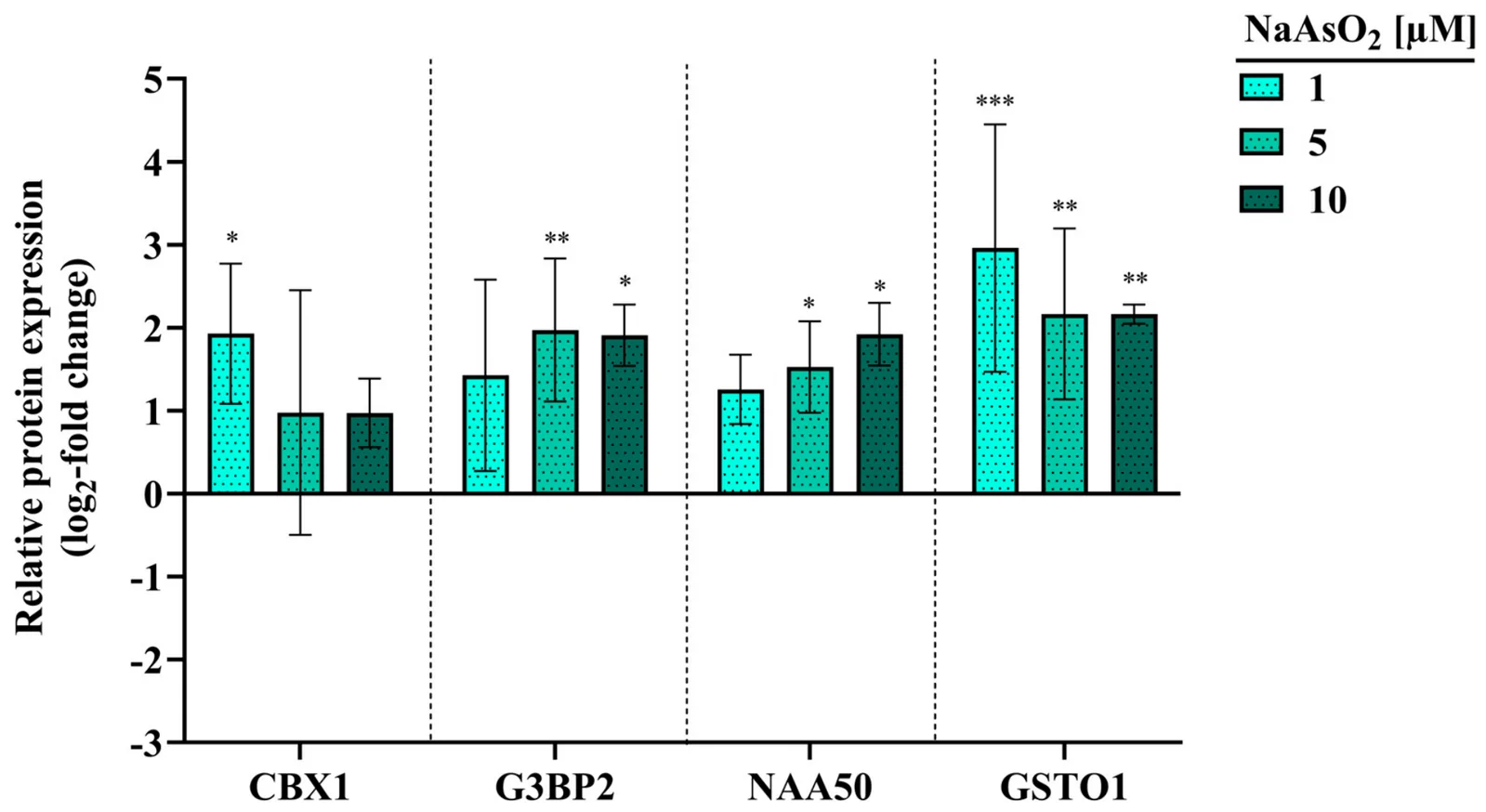
Effects of NaAsO_2_ on the expression of selected proteins. BEAS-2B cells were incubated with arsenite for 24 h. Relative protein expression is shown as mean ± SD of three independent experiments. each comprising triplicate parallel cell cultures and one LC-MS injection per sample. A two-way ANOVA with a Dunnett’s post hoc test was performed for statistical analysis compared to the untreated controls (* *p* ≤ 0.05, ** *p* ≤ 0.01, *** *p* ≤ 0.001).

Following arsenite incubation, particularly proteins related to oxidative stress were significantly and concentration-dependently induced. The most pronounced effect was the induction of HMOX1, which reached 38-fold expression levels at the lowest concentration of 1 µM arsenite. This corresponds to the gene expression data described above; however, the effects at the protein level were more pronounced than those at the gene expression level. The relative expression levels of four selected proteins, such as chromobox protein homolog 1 (CBX1), Ras GTPase-activating protein-binding protein 2 (G3BP2), N-acetyltransferase 50 (NAA50) and glutathione S-transferase omega-1 (GSTO1) after arsenite treatment are shown in Figure 9. GSTO1 protein expression increased approximately eightfold compared to controls at 1 µM arsenite. GSTO1 exhibits MMA^5+^ reductase activity and is therefore part of the cellular arsenic biotransformation machinery [49]. Consequently, its induction by arsenite suggests potentially active arsenic metabolism in BEAS-2B cells. However, a decrease in *GSTO1* gene expression was observed in rat kidney cortices exposed to up to 10 µg/mL of arsenite in drinking water for 10 days [50]. These results suggest that GSTO1 expression is time- and cell line-dependent following arsenite treatment, even at non-cytotoxic concentrations.

CBX1 protein expression increased about 3.8-fold following treatment with 1 µM arsenite, compared to the control samples. NAA50 protein expression increased 2.4-fold at the lowest incubation concentration, and continued to increase in a concentration-dependent manner. Both CBX1 and NAA50 are involved in chromatin remodeling and interact with histone proteins [51,52]. Previous studies have described the impact of arsenite on epigenetic mechanisms, transcriptional regulation, and histone modifications [53], but, to our knowledge, the impact on these specific proteins has not yet been reported.

Additionally, G3BP2 protein expression increased significantly after treatment with 5 µM and 10 µM arsenite. This protein plays a role in forming cellular stress granules. The formation of these particles is a known mechanism following arsenite treatment [54] and is supported by these data.

**Figure 10:**
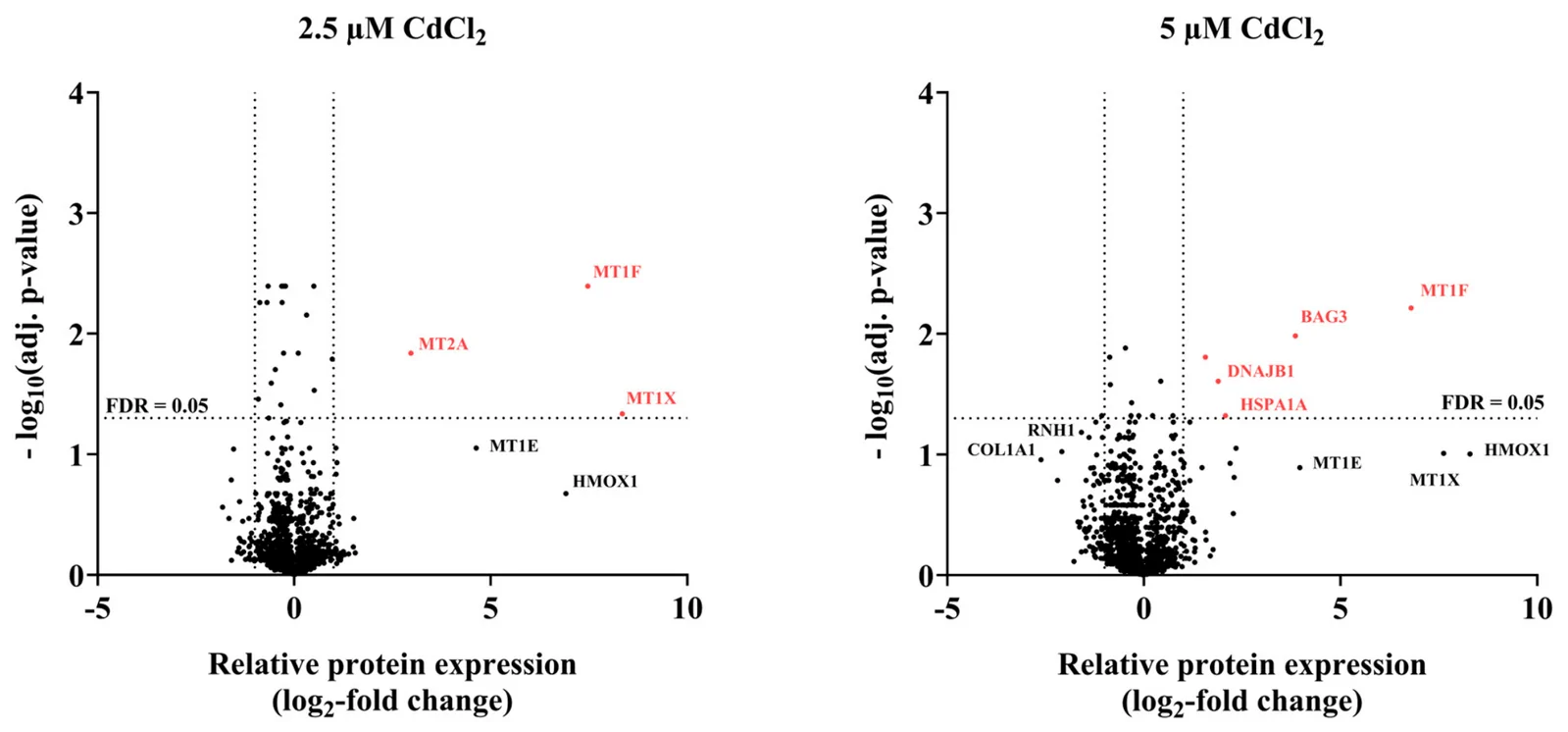
Impact of CdCl_2_ on the protein expression. BEAS-2B cells were incubated with 2.5 µM and 5 µM CdCl_2_ for 24 h. The relative protein expression is shown as the mean of three independent experiments. each comprising triplicate parallel cell cultures and one LC-MS injection per sample. Statistical significance compared to untreated controls was assessed by a two-sided Student’s *t*-test with Benjamini–Hochberg correction for multiple testing (false discovery rate (FDR) ≤ 0.05). Proteins showing significant and relevant changes in abundance are highlighted in blue when reduced by at least 50% and in red when increased at least two-fold relative to the control. Black dots indicate no significant and/or relevant changes in protein expression.

Consistent with the gene expression analysis, a pronounced MT induction was observed at the protein level after 24 h of cadmium exposure. Notably, MTF1 showed the most significant effect in relative protein expression (Figure 10). Unlike arsenite, no significant HMOX1 induction was observed, despite gene expression changes of up to 120- and 313-fold after exposure to 2.5 µM and 5 µM cadmium, respectively.

Both metal compounds induced the expression of cellular stress-related proteins and chaperones, including the BAG family molecular chaperone regulator 3 (BAG3), at the highest incubation concentration. BAG3 expression is regulated by heat shock factor 1 (HSF-1) [55]. Heat shock 70 kDa protein 1A (HSPA1A) and the co-chaperones DNAJA1, DNAJB1, and DNAJB4 were induced by both metals in a concentration-dependent manner. Interestingly, other members of these protein families, such as DNAJA2 and DNAJC9, were not induced by arsenite or cadmium. DNAJ proteins are classified into three types based on the presence of specific structural domains. DNAJ proteins interact with HSP70 and can influence its function due to their high structural divergence [56]. A previous study with T cells reported substantial increases in the gene expression of *DNAJA1*, *DNAJB1*, and *DNAJB4* following heat stress. However, the expression of *DNAJA2* and *DNAJC9* was not or only slightly induced [57]. These data suggest that treatment with arsenite and cadmium induces heat-stress signaling in BEAS-2B cells. The expression of heat shock proteins, including DNAJ proteins, is primarily regulated by the transcription factor HSF-1. In lymphoblast cells, 15 out of 17 DNAJ proteins were identified as HSF-1-regulated, including DNAJA1 and DNAJB1. Interestingly, DNAJB4 was strongly induced after heat shock, despite the absence of HSF-1 binding [58]. Thus, the cellular heat shock response may be induced by protein damage or aggregation in response to various stimuli, including oxidative stress [59]. Under basal non-stress conditions, HSF-1 is typically found in a complex with other chaperones. If misfolding occurs, the chaperones dissociate from the complex, move to the affected sites, and finally activate HSF-1 [60].

Overall, treatment of keratinocytes with 5 µM arsenite resulted in a similar profile of induced protein expression, as determined by SILAC and subsequent in-gel digestion [19]. Our data revealed even more detailed effects on specific proteins. In a recent study, Zhao et al. used iTRAQ labeling to analyze protein expression after treating BEAS-2B cells with 2.5 and 5 µM arsenite. Interestingly, this study demonstrated that arsenite leads to nearly complete suppression of protein expression [20], a finding that cannot be confirmed by our data.

### 2.5. Comparison of Gene and Protein Expression Analysis

**Figure 11:**
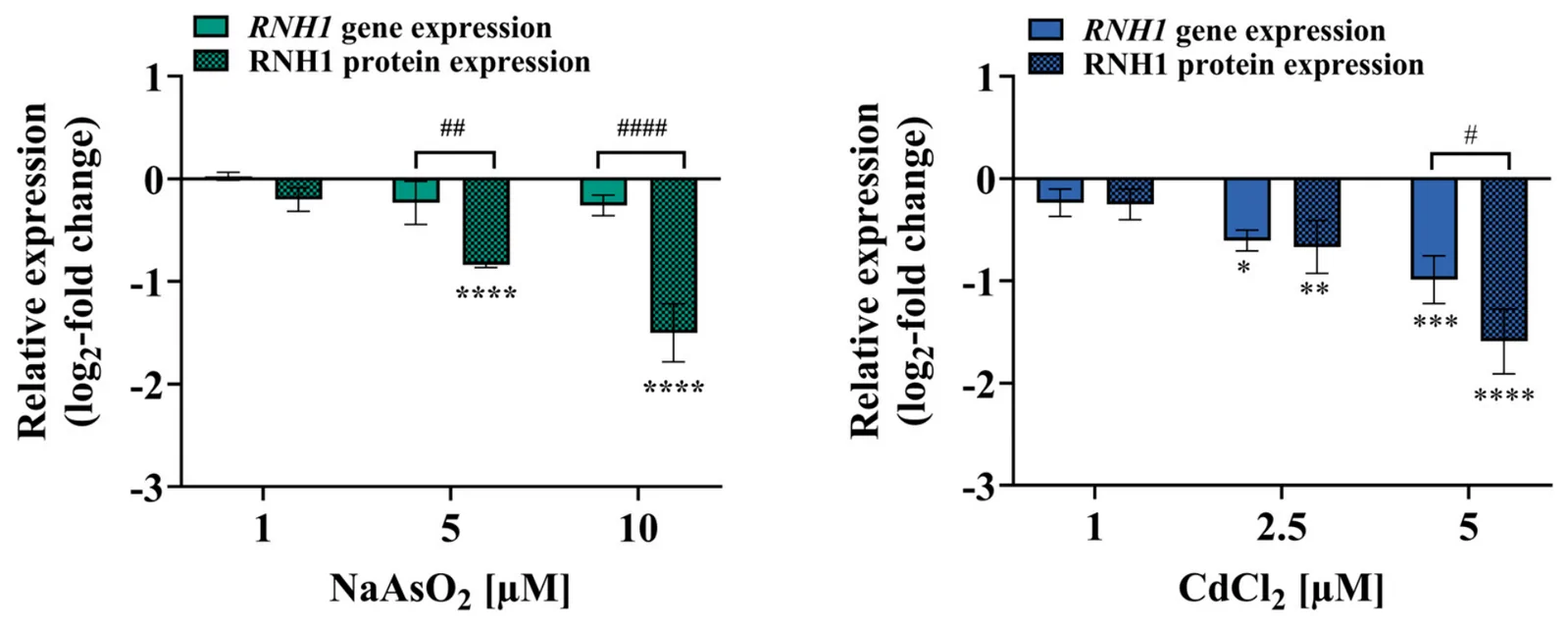
Comparison of gene- and protein expression of ribonuclease inhibitor 1 (RNH1) after 24 h NaAsO_2_ and CdCl_2_ incubation in BEAS-2B cells. Relative gene and protein expression is shown as the mean ± SD of three independent experiments. A two-way ANOVA with a Tukey’s multiple comparison test was performed for statistical analysis, comparing the results to the untreated controls (* *p* ≤ 0.05, ** *p* ≤ 0.01, *** *p* ≤ 0.001, **** *p* ≤ 0.0001) and comparing the gene and protein expression (# *p*≤ 0.05, ## *p* ≤ 0.01, #### *p* ≤ 0.0001).

In general, the data obtained at the mRNA and protein levels are mostly comparable. However, significant differences were detected in a few cases. One such example is the ribonuclease inhibitor 1 (RNH1) (Figure 11). Exposure to 5 µM or 10 µM of NaAsO_2_ for 4 h reduced RNH1 protein expression by 44% and 65%, respectively. Similar effects were observed after CdCl_2_ exposure. Notably, *RNH1* gene expression was not significantly altered by arsenite exposure. In contrast, mRNA levels decreased by 35% and 50% at the middle and highest cadmium incubation concentrations. A statistically significant difference in relative mRNA and protein levels was observed at 5 and 10 µM NaAsO_2_ and at 5 µM CdCl_2_.

RNH1 is a cytoplasmic protein rich in leucine and cysteine residues. Under physiological conditions, it binds to different RNases, thereby playing a role in regulating the cell cycle and survival. RNH1 is susceptible to oxidative stress and easily becomes oxidized. This can alter protein conformation and, consequently, lead to its degradation by the cellular proteasome [61,62]. Previous studies in mouse embryonic fibroblasts support this hypothesis. One study showed that incubating cells for two hours with a very high concentration of 500 µM NaAsO_2_ resulted in a loss of cellular RNH1 protein. In contrast, gene expression remained almost unaffected [63]. Therefore, oxidative stress following incubation with arsenite or cadmium may explain RNH1 degradation. However, a concentration-dependent decrease in mRNA levels was detected for cadmium, suggesting that the transcription of RNH1 may also be affected. Overall, our study confirms RNH1 degradation even at sub-cytotoxic concentrations. This could lead to an increase in free RNases and affect genomic stability.

The results clearly show that changes in gene expression do not necessarily correspond to changes in protein expression profiles. Besides protein degradation, mRNA stability is considered a major underlying factor of differences in expression profiles at a given time point [64]. A previous study showed higher mRNA levels of selected genes after eight hours of cadmium treatment than after 24 h in BEAS-2B cells [15]. More insights could be gained by performing time-dependent analyses. Another possible explanation is the impact of cadmium on protein folding. Low concentrations of cadmium have recently shown to cause misfolding and denaturation of newly synthesized, especially short-lived proteins, followed by proteasomal degradation in human lung and kidney cells. This may be explained by Cd(II) binding to critical SH groups [65].

## 3. Materials and Methods

### 3.1. Materials

All chemicals were purchased from Carl Roth (Karlsruhe, Germany), VWR (Radnor, PA, USA), Thermo Fisher Scientific (Waltham, MA, USA), Corning (Corning, NY, USA), or Sigma-Aldrich (Burlington, VT, USA) at a minimum purity of 99%. Cell culture and PCR consumables were obtained from Sarstedt (Nümbrecht, Germany), Brand (Wertheim, Germany), and Eppendorf (Hamburg, Germany). Cell culture media and additives were purchased from Lonza (Basel, Switzerland). The chemicals and materials for PCR analysis were obtained from Standard Biotools (San Francisco, CA, USA), Applied Biosystems (Waltham, MA, USA), Teknova (Hollister, CA, USA), New England Biolabs (Ipswich, MA, USA), Biotium (Fremont, CA, USA), BioCat (Heidelberg, Germany), and Bio-Rad (Hercules, CA, USA). Primers were synthesized by Eurofins (Hamburg, Germany). Atomic absorption spectroscopy consumables were purchased from PerkinElmer (Waltham, MA, USA). For proteomics analysis, HPLC vials were obtained from LLG Labware (Meckenheim, Germany), purification filters from Pall (Port Washington, NY, USA), and the reverse-phase C18 column from Phenomenex (Torrance, CA, USA).

### 3.2. Cell Culture and Metal Treatment

The human lung epithelial cell line BEAS-2B (ATCC CRL-3588) was kindly provided by Dr. Carsten Weiss (Karlsruhe Institute of Technology, Karlsruhe, Germany). The cells were used exclusively from passages 45 to 55. Prior to cultivation, the cells were pre-coated with a mixture of 10 µg/mL bovine fibronectin, 30 µg/mL rat tail collagen, and 10 µg/mL bovine serum albumin in PBS for at least 30 min at 37 °C. The cells were cultured in keratinocyte growth medium at 37 °C with 5% CO_2_ and 100% humidity, and subcultured once a week. For the cell culture experiments and metal treatment, logarithmically growing cells were incubated with NaAsO_2_ or CdCl_2_ for 24 h. Untreated control samples were maintained under the same conditions. For cellular uptake analysis, the incubation time was extended to 48 h.

### 3.3. ATP Content

To measure cytotoxicity, the CellTiterGlo^®^ Luminescent Cell Viability Assay Kit (Promega, Walldorf, Germany) was used to determine intracellular ATP levels. 3 × 10^4^ cells were seeded into 96-well cell culture plates. After incubation with metal compounds for 24 h, the medium was removed, and the cells were washed with PBS. The assay was performed according to the manufacturer’s protocol. In brief, 60 µL of medium was added to each well, and the plate was equilibrated at room temperature for 30 min. Then, 60 µL of the assay mix was added to each well. The luminescence signal was measured after a short period of orbital shaking and 10 min equilibration using a Tecan Infinite^®^ 200Pro (Tecan, Männedorf, Switzerland) microplate reader. Relative ATP content was calculated as a percentage of the control.

### 3.4. Cellular Uptake

1.25 × 10^6^ cells were seeded into 60 mm cell culture plates and cultured for 24 h. The incubation times for NaAsO_2_ and CdCl_2_ ranged from one to 48 h. Subsequently, the cells were harvested, and the cell count and average cell volume were determined using a CASY^®^TT cell counter (OMNI Life Science, Bremen, Germany). The cell pellets were then washed with PBS, resuspended in 500 µL of an oxidative digestion solution (1:1 mixture of 69% HNO_3_ and 30% H_2_O_2_), and heated stepwise to 95 °C for 18 h. The residue was dissolved in 1 mL (Cd) or 250 µL (As) of 0.2% HNO_3_ by shaking for at least two hours. Cellular arsenic and cadmium levels were analyzed by GF-AAS (PinAAcle 900T, PerkinElmer, Rodgau, Germany). For arsenic measurements, an electrodeless discharge lamp at 193.7 nm was used. Cadmium was measured using a hollow-cathode lamp at a wavelength of 228.8 nm. Cellular metal content was calculated using a standard calibration ranging from 0 to 10 µg/L for arsenic and from 0 to 2.5 µg/L for cadmium. The temperature protocol is listed in Table S4. To validate the method, an external standard sample was measured for each independent experiment. The limits of decision, detection, and determination are listed in Table S5. Each value represents the average of three independent experiments, measured in duplicate with three technical replicates. Intracellular metal concentrations were calculated based on cell count and the average cell volume, as measured by the CASY^®^TT cell counter.

### 3.5. High Throughput-RT-qPCR

For the gene expression analysis, 1 × 10^6^ cells were cultured in 6 cm plates for 24 h, after which they were incubated with CdCl_2_ and NaAsO_2_ for an additional 24 h. After washing with PBS and harvesting the cells by treatment with Accutase^®^ for 4 min at 37 °C, the cell pellet was resuspended in ice-cold PBS. RNA isolation was performed using the MN NucleoSpin^®^ RNA Plus Kit (Macherey-Nagel, Düren, Germany) according to the manufacturer’s protocol. cDNA synthesis and the subsequent high-throughput workflow were performed as previously described, using a Dynamic Array BioMark^TM^ (Fluidigm Corporation, San Francisco, CA, USA) [15]. Overall, the expression of 95 genes was analyzed in 96 samples simultaneously. The original gene set (reported in [15,66]) was modified to include several genes involved in redox regulation and the oxidative stress response. The complete gene set and all newly established primer data are listed in Tables S6 and S7. The data were processed using Fluidigm RT Analysis and GenEx software (version 5.3.6.170). Five reference genes (*ACTB*, *B2M*, *GAPDH*, *GUSB*, and *HPRT1*) were used for data normalization. The results are presented as the log_2_-fold change relative to the untreated control samples, using the ΔΔCq method [67].

### 3.6. Shotgun Proteomics

#### 3.6.1. Filter-Aided Sample Preparation

To analyze protein expression, 1 × 10^6^ cells were cultured in 6 cm plates for 24 h. The cells were then incubated in triplicate with NaAsO_2_ or CdCl_2_ for 24 h. After washing and harvesting, the cell pellet was washed three times with 1 mL of ice-cold PBS. To efficiently prepare the samples, Nanosep^TM^ 10 K microcentrifugation filters (Pall, Port Washington, NY, USA) were used to purify the proteins for LC-MS/MS analysis. The sample preparation workflow was performed according to a previously published protocol [68], with minor modifications. For protein extraction, the cells were incubated on ice in lysis buffer (7 M urea, 2 M thiourea, 5 mM DTT, and 2% CHAPS) for 15 min. Then, the solution was sonicated for 30 s with 10 s pulses at 10% amplitude using a Branson ultrasonicator disruptor horn (Heinemann, Schwäbisch Gmünd, Germany). To remove cell debris, the solution was centrifuged at 20,000× *g* for 15 min. Next, the protein concentration was measured using the Pierce^TM^ 660 nm Assay (Thermo Fisher Scientific, Waltham, MA, USA) according to the manufacturer’s instructions.

The filters were preconditioned with 1% formic acid (FA). After every incubation or washing step, the filters were centrifuged for 15 min at 12,000× *g*. Then, 20 µg of protein per sample was loaded onto the microcentrifugal filters. Washing steps were performed with urea buffer (8 M urea, 100 mM Tris, pH 8.5) followed by centrifugation. The proteins were reduced with 8 mM DTT for 15 min at 56 °C with gentle shaking. Afterward, the proteins were washed with urea buffer and alkylated using a 50 mM IAM solution for 20 min at room temperature. After another washing step, the excess IAM was quenched with 8 mM DTT for 15 min at 56 °C with gentle shaking. After the final wash, the filters were centrifuged for 20 min to remove any remaining solution. To perform a buffer exchange, the filters were washed three times with a 50 mM ammonium bicarbonate (ABC) buffer solution. After the final washing step, the filters were placed in low-protein-binding tubes. For protein digestion, 0.01 µg/µL trypsin in ABC buffer was loaded onto each filter. The filters were then incubated overnight at 37 °C in a humidified incubator. Peptides were then eluted by centrifugation at 12,000× *g* for 10 min. This step was repeated with 40 µL of ABC buffer. The peptide solution was then acidified with TFA to reach a final TFA concentration of 0.2%. Finally, the samples were evaporated to complete dryness.

#### 3.6.2. Shotgun Proteomics: LC-MS/MS

The dried peptides were dissolved in 50 µL of LC-MS-grade water containing 2% acetonitrile (ACN) and 0.1% FA (*v*/*v*). The peptide concentration was determined at 205 nm using a NanoDrop^TM^ One spectrophotometer (Thermo Fisher Scientific, Waltham, MA, USA). Proteomics analysis was performed using an UltiMate^TM^ 3000 RSLCnano system coupled to an Orbitrap Q Exactive^TM^ Plus MS/MS system (Thermo Fisher Scientific, Waltham, MA, USA). The solvents used were 0.1% FA in water (A) and 0.1% FA in ACN (B). The injection volume was calculated for 200 ng of peptides. First, increased purification was achieved using a trapping column with a flow rate of 8 µL/min and 100% solvent A for 5 min. Then, the sample was loaded onto a BioZenPeptide XB-C18, 2.6 μm, 250 μm × 0.075 mm analytical column (Phenomenex, Aschaffenburg, Germany) with a flow rate of 0.3 µL/min and a temperature of 40 °C. The linear gradient was as follows: 2% B at 0 min, 34% B at 60 min, 100% B from 62 to 65 min, and 2% B from 66 to 80 min. The analytical column was coupled directly to a Nanospray Flex^TM^ ion source for peptide ion generation. The ion spray voltage was set to 1.7–2.0 kV, the capillary temperature to 250 °C, and the S-lens level to 60.

The data were generated using a Top20 data-dependent acquisition (DDA) approach in a positive electrospray ionization mode. The isolation width was set to 2 *m*/*z*, the normalized collision energy to 28%, and the charge state to +2. For a full scan at the MS1 level, the resolution was set to 70,000, the automated gain control (AGC) to 3 × 10^6^, the scan range to 360–1300 *m*/*z*, and the maximum injection time to 50 ms. For MS2 scans, only ions with a charge of +2 to +7 were analyzed. The resolution was set to 17,000, the AGC target to 1 × 10^5^, and the maximum injection time to 50 ms. The MS^2^ spectra were recorded with a fixed first mass of 140 *m*/*z* and a dynamic exclusion time of 15 s. The total method runtime was 80 min.

#### 3.6.3. Shotgun Proteomics: Protein Identification and Data Processing

The FreeStyle software (version 1.8 SP, Thermo Fisher Scientific, Waltham, MA, USA) was used to visualize the mass spectra. MaxQuant software (version 2.4.2.0) was used to identify peptides and proteins from the human proteome. The human proteome data were obtained from the UniProt database (FASTA file, downloaded 25 October 2021; UP000005640) [69,70]. As fixed modifications, carbamidomethylation on cysteine (+57.02), and as variable modifications, oxidation of methionine (+15.99) and N-terminal protein acetylation (+42.01) were set. Intensity-based absolute quantification (IBAQ) was used for label-free quantification. For peptide and protein identification, the peptide spectrum match (PSM) and protein false discovery rates (FDR) were set to ≤0.01. After the MaxQuant calculation, the IBAQ values from each sample were summarized and normalized to a control sample using a normalization factor. Additionally, proteins identified only by site, reverse-identified proteins, and common contaminants were removed. The software Perseus (version 2.0.10.0) was used for further data processing [69]. The data were filtered to include only proteins with IBAQ values in at least two of the three parallel cell cultures. Missing values were imputed from a normal distribution with a width of 0.3 and a downshift of 1.8.

### 3.7. Statistical Analysis

All endpoints were determined in three independent experiments. Each of them was carried out in duplicate parallel cell cultures (gene expression, cellular uptake) or in triplicate parallel cell cultures (cytotoxicity, shotgun proteomics). Gene expression was measured in two technical replicates per sample, and cellular uptake experiments were performed in three technical replicates. All results are presented as the mean from at least three independent experiments ± standard deviation. The number of parallel determinations and measurements is described in the respective figure legends. Statistical analyses were performed using GraphPad Prism (version 10.4.1). One- or two-way ANOVA followed by Dunnett’s or Tukey’s multiple comparison test was used to analyze statistical differences between samples and the untreated controls; *p*-values ≤ 0.05 were considered statistically significant. The statistical evaluation of the differential protein expression of all identified proteins in shotgun proteomics depicted as a volcano plot was assessed using a two-sided Students’ *t*-test in Perseus. To account for multiple hypothesis testing, *p*-values were adjusted (adj.) using the Benjamini–Hochberg false discovery rate (FDR) procedure in GraphPad Prism, with a threshold set to FDR ≤ 0.05.

## 4. Conclusions

This study aimed to gain further mechanistic insight into the cellular responses to non-cytotoxic and low-cytotoxic concentrations of cadmium or arsenite in BEAS-2B cells. Our data showed that cadmium is more toxic and accumulates more efficiently in the cells than arsenite. At comparable cytotoxic concentrations, both metal compounds modulated the expression of genes and proteins associated with the activation of the redox-sensitive NRF2-dependent signaling pathway. After 24 h, this led to the expression of oxidative stress and antioxidant response genes, as well as their respective proteins. Additionally, we demonstrated that the ER stress response and protein folding were impacted at higher concentrations. In our study, arsenite affected the expression of selenoprotein- and ferroptosis-associated genes more pronounced than cadmium did. In contrast, cadmium strongly induced genes and proteins related to metal homeostasis and inflammatory signaling cascades. Notably, specific effects were also observed at low incubation concentrations. At the protein level, factors involved in chromatin remodeling and RNA metabolism were affected. However, further mechanistic studies are required to identify specific interactions, such as those with cysteine residues involved in cellular redox regulation.

## Data Availability

The original contributions presented in this study are included in the Supplementary Material. The mass spectrometry proteomics data have been deposited to the ProteomeXchange Consortium via the PRIDE [71] partner repository with the dataset identifier PXD076643 (Token for review: 6RS7e2VhhYKT). Further inquiries can be directed to the corresponding author.

## Supplementary Materials

The following supporting information can be downloaded at https://www.mdpi.com/article/10.3390/ijms27177809/s1.

## Abbreviations

The following abbreviations are used in this manuscript:

ABC	Ammonium bicarbonate
AGC	Automated gain control
DDA	Data-dependent acquisition
DMA	Dimethylarsinic acid
FDR	False discovery rate
GF-AAS	Graphite furnace atomic absorption spectroscopy
GSH	Glutathione
HSF-1	Heat shock factor 1
IBAQ	Intensity-based absolute quantification
iTRAQ	Isobaric tags for relative and absolute quantitation
MMA	Monomethylarsonic acid
MT	Metallothionein
MTF-1	Metal regulatory transcription factor 1
NF-κB	Nuclear factor ‘kappa-light-chain-enhancer’ of activated B-cells
NRF2	Nuclear factor erythroid 2-related factor 2
SD	Standard deviation
SILAC	Stable isotope labeling by amino acids in cell culture
UPR	Unfolded protein response
