# Supplementary material for: Distinct Interactions of Arsenite and Cadmium with Metal Homeostasis and the Cellular Stress Response in Human Bronchial Epithelial Cells
Source: Int J Mol Sci. 2026 Aug 31;27(17):7809. doi: 10.3390/ijms27177809 (PMC13566881; doi:10.3390/ijms27177809)
Supplement: Supplementary file 1 [file ijms-27-07809-s001.zip › ijms-4494469-supplementary.pdf]

International Journal of
Molecular Sciences

MDPI

Article

Distinct interactions of arsenite and cadmium with metal homeostasis and the cellular stress response in human bronchial epithelial cells

Martin Link, Jana Kuhn, Marlene Parsdorfer and Andrea Hartwig *

Department of Food Chemistry and Toxicology, Institute of Applied Biosciences (IAB), Karlsruhe Institute of Technology (KIT), 76131 Karlsruhe, Germany

* Correspondence: andrea.hartwig@kit.edu

Table S1: Gene set with genes and their respective encoded proteins used in this study.

	Gene	Encoded protein
**Reference genes**	*ACTB*	Actin, cytoplasmic 1
	B2M	Beta-2-microglobulin
	GAPDH	Glyceraldehyde-3-phosphate dehydrogenase
	GUSB	Beta-glucuronidase
	HPRT1	Hypoxanthine-guanine phosphoribosyltransferase
**RNAse**	*RNASEH2A*	Ribonuclease H2 subunit A
	RNH1	Ribonuclease H1
**Xenobiotic metabolism**	*NQO1*	NAD(P)H dehydrogenase [quinone] 1
	SULT1A1	Sulfotransferase 1A1
**Inflammation**	*ANXA1*	Annexin A1
	IL1A	Interleukin-1 alpha
	IL8	Interleukin-8
	PDGFA	Platelet-derived growth factor subunit A
	TGFb	Transforming growth factor beta-1 proprotein
**Metal homeostasis**	*FTH1*	Ferritin heavy chain
	MT1X	Metallothionein-1X
	MT2A	Metallothionein-2
	SLC30A1	Proton-coupled zinc antiporter SLC30A1
	TFRC	Transferrin receptor protein 1
**Redox regulation and oxidative stress response**	*APP*	Amyloid-beta precursor protein
	BAG2	BAG family molecular chaperone regulator 2
	CAT	Catalase
	DNAJC17	DnaJ homolog subfamily C member 17
	ETHE1	Persulfide dioxygenase ETHE1, mitochondrial
	G6PD	Glucose-6-phosphate 1-dehydrogenase
	GCLC	Glutamate--cysteine ligase catalytic subunit
	GCLM	Glutamate--cysteine ligase regulatory subunit

 https://doi.org/10.3390/ijms27177809

	GLRX	Glutaredoxin-1
	GLRX2	Glutaredoxin-2, mitochondrial
	GPX1	Glutathione peroxidase 1
	GPX2	Glutathione peroxidase 2
	GPX4	Phospholipid hydroperoxide glutathione peroxidase GPX4
	GSR	Glutathione reductase, mitochondrial
	GSTP1	Glutathione S-transferase P
	HMOX1	Heme oxygenase 1
	HSPA1A	Heat shock 70 kDa protein 1A
	LONP1	Lon protease homolog, mitochondrial
	MSRB3	Methionine-R-sulfoxide reductase B3
	NXN	Nucleoredoxin
	PARK7	Parkinson disease protein 7
	PDIA4	Protein disulfide-isomerase A4
	PEX5	Peroxisomal targeting signal 1 receptor
	PRDX1	Peroxiredoxin-1
	PRDX6	Peroxiredoxin-6
	PRXL2A	Peroxiredoxin-like 2A
	PXDNL	Probable oxidoreductase PXDNL
	SELENOF	Selenoprotein F
	SELEONP	Selenoprotein P
	SELT	Thioredoxin reductase-like selenoprotein T
	SOD1	Superoxide dismutase [Cu-Zn]
	SOD2	Superoxide dismutase [Mn], mitochondrial
	SQOR	Sulfide:quinone oxidoreductase, mitochondrial
	SQSTM1	Sequestosome-1
	STIP1	Stress-induced-phosphoprotein 1
	TMX1	Thioredoxin-related transmembrane protein 1
	TMX2	Thioredoxin-related transmembrane protein 2
	TXN	Thioredoxin
	TXN2	Thioredoxin, mitochondrial
	TXNDC12	Thioredoxin domain-containing protein 12
	TXNDC17	Thioredoxin domain-containing protein 17
	TXNRD1	Thioredoxin reductase 1, cytoplasmic
	TXNRD3	Thioredoxin reductase 3
**Signaling**	*AKAP2*	A-kinase anchor protein 2
	AKAP12	A-kinase anchor protein 12
	AKAP13	A-kinase anchor protein 13
	ATOX1	Copper transport protein ATOX1
	CCDC50	Coiled-coil domain-containing protein 50
	EGFR	Epidermal growth factor receptor
	JUN	Transcription factor Jun

https://doi.org/10.3390/ijms27177809

	KEAP1	Kelch-like ECH-associated protein 1
	MAP3K5	Mitogen-activated protein kinase kinase kinase 5
	MYC	Myc proto-oncogene protein
	NFE2L2	Nuclear factor erythroid 2-related factor 2
	NFKB1	Nuclear factor NF-kappa-B p105 subunit
	NFKB2	Nuclear factor NF-kappa-B p100 subunit
	NFKBIA	NF-kappa-B inhibitor alpha
	NNT	NAD(P) transhydrogenase, mitochondrial
	NOX4	NADPH oxidase 4
	PLK3	Serine/threonine-protein kinase PLK3
**Cell cycle and apoptosis**	*BAX*	Apoptosis regulator BAX
	BCL2	Apoptosis regulator Bcl-2
	CCND1	G1/S-specific cyclin-D1
	CDKN1A	Cyclin-dependent kinase inhibitor 1
	CDKN1B	Cyclin-dependent kinase inhibitor 1B
	CDKN2B	Cyclin-dependent kinase 4 inhibitor B
	GAB1	GRB2-associated-binding protein 1
	HUWE1	E3 ubiquitin-protein ligase HUWE1
	MDM2	E3 ubiquitin-protein ligase Mdm2
	RIPK3	Receptor-interacting serine/threonine-protein kinase 3
	TP53	Cellular tumor antigen p53
**DNA repair**	*APEX1*	DNA repair nuclease/redox regulator APEX1
	MLH1	DNA mismatch repair protein Mlh1
	MSH2	DNA mismatch repair protein Msh2
	MSH6	DNA mismatch repair protein Msh6
	PARP1	Poly [ADP-ribose] polymerase 1

https://doi.org/10.3390/ijms27177809

Table S2: Gene expression results using a high-throughput RT-qPCR approach. BEAS-2B cells were incubated with $NaAsO_2$ and $CdCl_2$ for 24 h. Shown are the mean values out of three independent experiments as log_2 fold change. For each experiment, the cell culture treatment and sample preparation were performed in duplicate. For PCR alanysis, each sample was measured twice.

log_2 fold change		$NaAsO_2$			$CdCl_2$		
		1 µM	5 µM	10 µM	1 µM	2.5 µM	5 µM
**RNAse**	*RNASEH2A*	-0.3	-0.7	-0.9	-0.1	-0.4	-0.6
	RNH1	0.0	-0.2	-0.3	-0.2	-0.6	-1.0
**Xenobiotic metabolism**	*NQO1*	0.6	1.0	1.0	0.5	0.9	0.9
	SULT1A1	0.0	-0.4	-0.6	0.1	-0.3	-0.3
**Inflammation**	*ANXA1*	0.1	0.2	-0.3	0.1	0.2	1.6
	IL1A	0.5	0.9	1.7	1.1	3.9	5.4
	IL8	-0.1	-0.1	0.6	1.4	4.7	8.0
	PDGFA	0.2	0.1	0.1	0.4	3.8	4.3
	TGFb	-0.2	-0.6	-0.6	-0.1	-0.2	-0.5
**Metal homeostasis**	*FTH1*	0.7	1.3	1.3	0.7	0.8	1.4
	MT1X	0.3	2.7	3.0	5.6	6.0	6.9
	MT2A	0.4	2.0	2.0	2.7	2.7	2.7
	SLC30A1	0.2	0.5	0.7	1.1	1.7	3.8
	TFRC	0.6	0.9	1.1	1.1	1.6	0.9
**Redox regulation and oxidative stress response**	*APP*	0.3	0.4	0.5	0.0	0.5	0.7
	BAG2	0.2	0.3	0.4	0.1	0.3	0.5
	CAT	0.0	-0.2	-0.5	-0.1	-0.5	-1.2
	DNAJC17	0.1	0.1	0.0	-0.1	-0.3	-0.8
	ETHE1	-0.3	-0.6	-0.6	0.0	-0.2	-0.1
	G6PD	0.2	0.1	0.0	0.3	0.4	0.1
	GCLC	0.3	0.5	0.6	0.0	0.3	1.1
	GCLM	0.9	1.6	2.0	0.7	2.3	3.0
	GLRX	0.1	0.2	0.1	0.2	0.7	1.5
	GLRX2	0.1	0.3	0.5	0.1	0.1	-0.3
	GPX1	-0.1	-0.6	-1.2	0.0	-0.2	-0.2
	GPX4	-0.3	-0.5	-0.8	0.0	-0.3	-0.1
	GSR	0.3	0.1	0.1	-0.1	0.1	-0.2
	GSTP1	-0.1	-0.1	-0.1	0.1	-0.1	-0.2
	HMOX1	2.4	5.2	5.9	2.5	5.8	7.2
	HSPA1A	0.1	0.1	0.2	1.0	3.6	5.2
	LONP1	0.1	-0.1	-0.4	-0.1	-0.1	-0.6
	MSRB3	0.1	0.1	0.1	-0.2	-0.3	-0.5
	NXN	0.2	0.0	-0.1	-0.3	-0.5	-0.7
	PARK7	-0.4	-0.5	-0.6	0.1	0.0	0.0
	PDIA4	0.0	-0.9	-1.7	0.0	0.3	0.6
	PEX5	0.0	0.2	0.2	-0.1	-0.3	-0.7

	PRDX1	0.0	0.3	0.4	0.2	0.4	0.4
	PRDX6	-0.2	-0.2	-0.3	0.1	-0.1	-0.1
	PRXL2A	-0.1	-0.4	-0.5	0.1	-0.2	-1.1
	SELENOF	0.0	0.0	-0.2	0.0	-0.2	0.1
	SELEONP	-0.1	-0.9	-1.1	-0.1	-0.8	-1.2
	SELT	0.0	-0.1	-0.3	0.1	0.0	-0.4
	SOD1	0.0	0.2	0.3	0.1	0.0	-0.1
	SOD2	0.1	0.0	0.2	0.1	0.4	0.5
	SQOR	0.0	-0.3	-0.5	0.1	0.0	-0.1
	SQSTM1	0.5	0.7	1.1	0.1	0.7	1.2
	STIP1	0.0	0.1	0.0	0.0	0.3	0.9
	TMX1	0.0	-0.3	-0.3	-0.1	-0.1	-0.8
	TMX2	0.1	0.2	0.1	-0.2	-0.1	-0.3
	TXN	-0.3	-0.1	0.1	0.4	0.5	0.4
	TXN2	-0.1	-0.2	-0.3	0.0	-0.2	-0.2
	TXNDC12	0.0	0.0	-0.1	0.0	0.1	0.0
	TXNDC17	-0.1	-0.2	-0.5	0.1	-0.2	-0.5
	TXNRD1	0.6	0.9	1.2	0.2	1.4	3.0
	TXNRD3	0.3	-0.2	-0.6	-0.2	-0.5	-1.6
**Signaling**	*AKAP2*	-0.1	-0.2	0.1	-0.1	0.3	1.1
	AKAP12	0.2	0.4	0.4	0.0	1.0	2.7
	AKAP13	0.1	-0.2	-0.2	0.0	-0.2	-1.0
	ATOX1	-0.1	0.0	-0.1	0.1	0.0	0.1
	CCDC50	0.0	0.0	0.2	0.0	0.1	0.5
	EGFR	0.2	-0.1	-0.2	-0.2	-0.1	0.4
	JUN	0.3	0.3	0.6	0.1	1.3	2.9
	KEAP1	0.1	0.0	0.1	-0.1	-0.3	-1.7
	MAP3K5	0.0	-0.3	-0.2	0.1	0.3	0.3
	MYC	0.5	0.8	1.1	0.1	0.9	1.9
	NFE2L2	-0.1	-0.4	-0.4	0.0	-0.2	-0.4
	NFKB1	0.1	0.0	0.0	0.0	-0.1	0.1
	NFKB2	0.2	0.4	0.5	0.1	0.3	0.0
	NFKBIA	0.1	-0.3	-0.7	-0.2	-0.8	-2.8
	NNT	0.1	-0.3	-0.5	-0.4	-0.7	-1.4
	PLK3	0.0	0.3	0.5	-0.2	0.2	1.0
**Cell cycle and apoptosis**	*BAX*	0.1	0.0	-0.2	-0.1	-0.1	-0.2
	BCL2	0.1	0.0	0.0	-0.2	-0.2	-0.5
	CCND1	0.5	1.3	1.4	0.1	1.0	1.7
	CDKN1A	0.1	0.0	-0.2	-0.3	0.0	1.3
	CDKN1B	0.1	-0.4	-0.5	-0.2	-0.8	-2.6
	CDKN2B	0.1	-0.6	-0.9	-0.3	-0.2	0.2
	GAB1	0.1	-0.3	-0.5	-0.3	-0.6	-1.8

https://doi.org/10.3390/ijms27177809

	HUWE1	0.2	-0.1	-0.2	-0.3	-0.5	-1.7
	MDM2	0.2	0.1	0.0	-0.3	-0.5	-1.4
	TP53	0.2	-0.1	-0.5	-0.3	-0.7	-1.5
**DNA repair**	*APEX1*	0.1	0.1	-0.2	0.0	-0.4	-0.8
	MLH1	0.1	-0.2	-0.5	-0.2	-0.4	-0.6
	MSH2	0.0	-0.3	-0.4	-0.1	-0.3	-0.8
	MSH6	0.0	-0.2	-0.3	0.0	-0.3	-2.0
	PARP1	0.2	-0.2	-0.4	-0.3	-0.5	-1.1

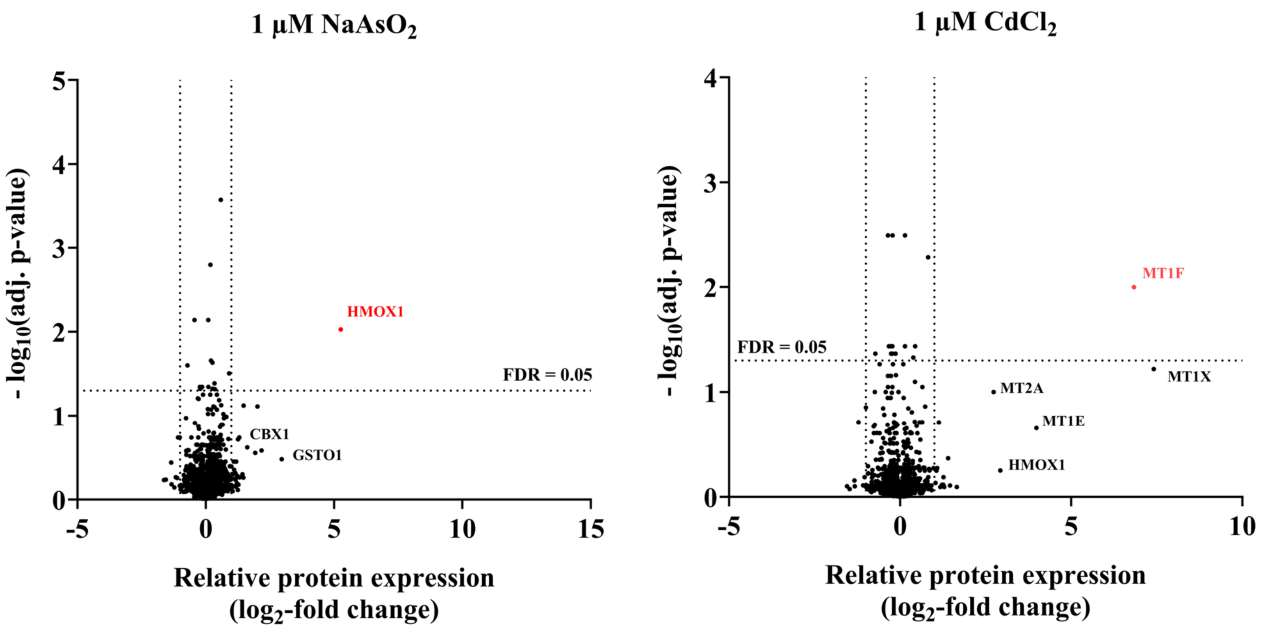

Figure S1: BEAS-2B cells were incubated with 1 µM $CdCl_2$ (left) and 1 µM $NaAsO_2$ (right) for 24 h. The relative protein expression is shown as the mean of three independent experiments, each comprising triplicate parallel cell cultures and one LC-MS injection per sample. Statistical significance compared to untreated controls was assessed by a two-sided Student's t-test with Benjamini–Hochberg correction for multiple testing (false discovery rate (FDR) ≤ 0.05). Proteins showing significant and relevant changes in abundance are highlighted in blue when reduced by at least 50% and in red when increased at least two-fold relative to the control. Black dots indicate no significant and/or relevant changes in protein expression

Table S3: Full protein names and UniProt identifiers for the proteins described in this study.

Gene	Encoded protein	UniProt ID
BAG3	BAG family molecular chaperone regulator 3	O95817
CBX1	Chromobox protein homolog 1	P83916
COL1A1	Collagen alpha-1(I) chain	P02452
G3BP2	Ras GTPase-activating protein-binding protein 2	Q9UN86
GSTO1	Glutathione S-transferase omega-1	P78417
HMOX1	Heme oxygenase 1	P09601
JUN	Transcription factor Jun	P05412
MSH6	DNA mismatch repair protein Msh6	P52701
MT1E	Metallothionein-1E	P04732
MT1F	Metallothionein-1F	P04733
MT1X	Metallothionein-1X	P80297
MT2A	Metallothionein-2	P02795
NAA50	N-alpha-acetyltransferase 50	Q9GZZ1
RNH1	Ribonuclease H1	O60930

Table S4: Decision, detection, and determination limit values of arsenic and cadmium based on DIN 32645 calculation in atomic absorption spectrometric experiments.

	**Arsenic**	**Cadmium**
Decision limit	0.168 µg/L	0.050 µg/L
Detection limit	0.335 µg/L	0.1 µg/L
Determination limit	0.937 µg/L	0.277 µg/L

https://doi.org/10.3390/ijms27177809

Table S5: Temperature program for atomic absorption spectrometric measurements of arsenic and cadmium.

**Arsenic**	Temperature [°C]	Ramp [s]	Holding time [s]	Argon [mL/min]
Drying	120	1	45	250
Drying	140	15	30	250
Pyrolysis	1200	10	20	250
Atomisation	2000	0	5	0
Bake out	2400	1	3	250
**Cadmium**				
Drying	120	1	45	250
Drying	140	15	30	250
Pyrolysis	500	10	20	250
Atomisation	1500	0	5	0
Bake out	2450	1	3	250

https://doi.org/10.3390/ijms27177809

Table S6: Primer sequences of newly designed primers for the redox homeostasis and oxidative stress response gene set (Ref = Reference sequence accession number; fw = forward; rv = reverse). All other primer sequences have already been described in Reference 15, Fischer et al., 2016.

Gene	Ref-Gene	Primer sequence 5' → 3' (fw)	Primer sequence 5' → 3' (rv)
AKAP12	NM_001370346	CCGAGATGGTTGGACAGAGA	GACTCAGTGGGTTGTGTTAGC
AKAP13	NM_001270546	AAGGAGGCGAGAAGGAAGAG	TCTGGACAACCTGCTCACT
AKAP2	NM_147150	GATGGCAGAGGCGGAATTG	CGAAGCACTTTGGACTTGGAAT
ANXA1	NM_000700	TCGCAGAGTGTTTCAGA	GAGGCATTTCTCAATGTCAC
APEX1	NM_001641	TTCTTACGGCATAGGCGATGA	AGGTCTCCACACAGCACAA
APP	NM_001136016	GCAGATGGGAGTGAAGACAAAG	CATCCTCATCGTCCTCGTCAT
ATOX1	NM_004045	CATGCCGAAGCACGAGTT	GTCAATGTCATACTTAACTCCTCCAAG
BAG2	NM_004282	GGCAGATCAGTGACGGAGAA	TGACCACCTCATCAATAATCCTTGT
CCDC50	NM_174908	GAGTCAAGGAAGTATGCCGAGAT	CTGCTTAGCCACCTGGAGAT
DNAJC17	NM_018163	AGGACCCAGAAACTTGATGAGAAA	GGCGTTCGATCTCTTGCTCTA
ETHE1	NM_001320869	CCTAAACCTCAGCAGATAGACTTTG	GCATTAATAGTGGATGGGAGCAT
GAB1	NM_002039	GATTCATCCTCTGCTACTCTACCT	ATGCGTTCTGGTGGGTTC
GCLM	NM_001308253	CACAGCGAGGAGGAGTTTC	GCCATGTCAACTGCACTTCTA
GLRX	NM_001118890	CCATCAAACAAGGGCTTCTGGAATT	TCGAGGCACCGTTCTTGCT
GLRX2	NM_197962	CCTGTGAACCAGATCCAAGAAAC	TATGAGTGTCAGTTGCACCTCC
GPX4	NM_002085	GCGGGCTACAACGTCAAAT	TGTCGATGAGGAACTTGGTGAA
HUWE1	NM_031407	GCTGCTAACTCGGCTACAAC	GCACTGGGTGGATATTTCATCAT
LONP1	NM_004793	ATTGCCTTGAACCCTCTCTACAG	GCCGCTTAGGAATATTGGTCTCT
MSH6	NM_001406831	GACTTGGTGCCTCAGACAGA	GCAGTACCTCTTCCTAATTCATCCA
MSRB3	NM_198080	TGCTCTCAGTGTGGTGCT	CACTTTGTTTCCATCACTCTCCAT
NNT	NM_001331026	GAGGAACTGTAGTGATGAAAGATGGTA	GCAGAAGCCGTTGACATTGT
NOX4	NM_001143836	AAGCAGGAGAACCAGGAGATT	GCCAGGAACAGTTGTGAAGAG
NXN	*NM_001205319*	GAAACCCTTCAGGGAAGTCATTG	CTCCGACCTGTCTGCACTAA
PARK7	NM_007262	CCAGTACAGTGTAGCCGTGAT	AGTAGGACCTGCACAGATGG
PDIA4	NM_001371244	TGAAGCAGGTCCAGGAGTT	CAGGTTGTTAGCGGCATCC
PEX5	NM_001374645	CCAAGTCAGCTATAGAGTCTGATGTC	CCGCAGCCCTTCTTCAAA
PRDX6	NM_004905	ACTTTCTGGGAGACTCATGGG	GGCAAGATGGTCCTCAACAC
PRXL2A	NM_001243782	CAGGAAAGCAGGGCATTCTTC	TTAGGGACACGAGTGGAAACAA
PXDNL	NM_144651	AGCACTCAGAGAGCAGATAAACA	ACTTGGCATTCCTCGGTCT
RIPK3	NM_006871	TGCCAACCGAACCATCACT	GGAAATCCTTTACCGTGGAGACA
RNASEH2A	NM_006397	GCGGAAGATGTTATATGGGAGGA	AGAGGCTGGTTGCTGACT
RNH1	NM_203388	GGTTAGCAACAACGACATCAATGA	CACAATGCCGCACAGGT
SELENOF	NM_203341	GTGCTTCAAGCGGTGTCTG	AACTGTCCGAGAAGATCACAAGAG
SELENOP	NM_005410	GACCTGCGAGTAAAACTGAAGAAAG	ACAAGACGGCCACATCTATCAT
SELT	NM_016275	TGAGGCTTCTGCTGCTTCT	CAGGAAACACAAATCTGGAACTTGA
SQOR	NM_021199	AAGGTTTCGCTCATCCCAAA	ATCGCTTCCCTGTCTTCCT
SQSTM1	NM_003900	TGCCCAGACTACGACTTGTGTA	TGCGAGAAGCCCTCAGACA
STIP1	NM_006819	ACCTGGGCACGAAACTACA	GCTCTGGCTTGGTCTCCTT

TMX1	NM_030755	CGGACTGTTATTAGGACTCTGTATGAT	GCGTTGTCTTATGGCATTCTGT
TMX2	NM_001144012	TCTATGCTGACCTCTCCCTTAAATACA	TTGCCTCCTTGCCACCTT
TXN2	NM_012473	GGATGGACCTGACTTTCAAGAC	ACCGCTGACACCTCATACT
TXNDC12	NM_015913	GCCGAGCAAGTTGTTCAGG	CCAGTGTAGGTAGGAATGAGGTT
TXNDC17	NM_032731	CAAGTAGGAGAAAAGCCTTATTGGA	TGAAGACACTCAGATTCTACCAGTT
TXNRD3	NM_001173513	AGGGTTCAAGAAGTGCTGTCA	CTCTGATATGCCTGGAAAGTTTGG

Table S7: Results of primer establishment (bp = base pair; T_m = melting temperature; R^2 = correlation coefficient; m = slope; E = primer efficiency).

Gene	bp (expected)	bp (determined)	T_m (expected)	T_m (determined)	R^2	m	E
AKAP12	184	170	80.2	81	0.9967	-3.269	1.02
AKAP13	132	120	78.1	79	0.9976	-3.270	1.02
AKAP2	141	132	80.2	79	0.9970	-3.264	1.02
ANXA1	94	69	75.2	78	0.9985	-3.261	1.03
APEX1	207	205	84.2	85	0.9989	-3.298	1.01
APP	100	89	78.7	80	0.9988	-3.235	1.04
ATOX1	103	101	79.4	82	0.9987	-3.227	1.04
BAG2	156	156	78.7	80	0.9994	-3.326	1.00
CCDC50	146	126	82.2	84	0.9992	-3.230	1.04
DNAJC17	139	118	84.2	86	0.9992	-3.276	1.02
ETHE1	110	82	80.0	83	0.9993	-3.251	1.03
GAB1	147	147	80.1	81	0.9990	-3.221	1.04
GCLM	158	150	76.7	78	0.9993	-3.270	1.02
GLRX	107	97	78.1	81	0.9993	-3.261	1.03
GLRX2	250	240	77.3	79	0.9985	-3.242	1.03
GPX4	154	135	82.6	84	0.9979	-3.286	1.02
HUWE1	111	102	78.8	81	0.9997	-3.276	1.02
LONP1	166	169	86.0	88	0.9987	-3.157	1.07
MSH6	133	103	77.1	79	0.9993	-3.288	1.01
MSRB3	205	216	84.2	86	0.9994	-3.347	0.99
NNT	163	169	79.9	81	0.9990	-3.244	1.03
NOX4	214	227	79.8	78	0.9967	-3.291	1.01
NXN	220	228	85.5	86	0.9994	-3.305	1.01
PARK7	204	195	82.3	83	0.9986	-3.257	1.03
PDIA4	113	98	81.1	82	0.9941	-3.100	1.1
PEX5	203	202	83.2	84	0.9991	-3.286	1.02
PRDX6	164	157	81.8	83	0.9979	-3.258	1.03
PRXL2A	208	212	79.8	79	0.9994	-3.267	1.02
PXDNL	241	254	84.9	85	0.9997	-3.335	0.99
RIPK3	249	273	84.4	86	0.9982	-3.253	1.03
RNASEH2A	154	139	83.1	84	0.9985	-3.217	1.05
RNH1	142	138	85.6	87	0.9957	-3.192	1.06
SELENOF	110	96	78.9	81	0.9876	-3.197	1.05
SELENOP	221	218	75.6	77	0.9989	-3.298	1.01
SELT	145	132	85.1	88	0.9979	-3.257	1.03
SQOR	192	195	80.2	81	0.9981	-3.234	1.04
SQSTM1	110	99	84.9	86	0.9950	-3.211	1.05
STIP1	144	128	83.6	84	0.9972	-3.287	1.01
TMX1	235	240	79.0	78	0.9983	-3.303	1.01
TMX2	162	147	80.7	82	0.9981	-3.159	1.07

TXN2	204	203	84.3	85	0.9991	-3.251	1.03
TXNDC12	214	193	80.5	81	0.9993	-3.304	1.01
TXNDC17	128	108	74.7	77	0.9984	-3.215	1.05
TXNRD3	110	101	75.5	78	0.9983	-3.304	1.01

https://doi.org/10.3390/ijms27177809
